# Efficacy and Safety of Pemafibrate, a Novel Selective Peroxisome Proliferator-Activated Receptor α Modulator (SPPARMα): Pooled Analysis of Phase 2 and 3 Studies in Dyslipidemic Patients with or without Statin Combination

**DOI:** 10.3390/ijms20225537

**Published:** 2019-11-06

**Authors:** Shizuya Yamashita, Hidenori Arai, Koutaro Yokote, Eiichi Araki, Mitsunori Matsushita, Toshiaki Nojima, Hideki Suganami, Shun Ishibashi

**Affiliations:** 1 Rinku General Medical Center, Osaka 598-8577, Japan; 2 National Center for Geriatrics and Gerontology, Aichi 474-8511, Japan; harai@ncgg.go.jp; 3 Department of Diabetes, Metabolism and Endocrinology, Chiba University Hospital, Chiba 260-8670, Japan; kyokote@faculty.chiba-u.jp; 4 Department of Endocrinology, Hematology and Gerontology, Chiba University Graduate School of Medicine, Chiba 260-8670, Japan; 5 Department of Metabolic Medicine, Faculty of Life Sciences, Kumamoto University, Kumamoto 860-8556, Japan; earaki@gpo.kumamoto-u.ac.jp; 6 Medical Affairs Department, Kowa Company, Ltd., Tokyo 103-8433, Japan; m-matust@kowa.co.jp; 7 Clinical Data Science Department, Kowa Company, Ltd., Tokyo 103-8433, Japan; t-nojima@kowa.co.jp (T.N.); suganami@kowa.co.jp (H.S.); 8 Division of Endocrinology and Metabolism, Department of Medicine, Jichi Medical University, Tochigi 329-0498, Japan; ishibash@jichi.ac.jp

**Keywords:** pemafibrate, triglyceride, selective peroxisome proliferator-activated receptor (PPAR)α modulator, renal dysfunction, lipoprotein subclass, high-performance liquid chromatography (HPLC), concomitant statin

## Abstract

Hypertriglyceridemia has emerged as an independent risk factor for cardiovascular events, despite low-density lipoprotein-cholesterol (LDL-C) well-controlled with statins. We pooled data from the first 12 weeks of six randomized double-blind placebo-controlled studies of pemafibrate in Japan and investigated its efficacy and safety with and without statins, particularly focusing on patients with renal dysfunction. Subjects were 1253 patients (677 in the “with-statin” group and 576 in the “without-statin” group). At Week 12 (last observation carried forward), triglyceride (TG) was significantly reduced at all pemafibrate doses (0.1, 0.2, and 0.4 mg/day), both with and without statin, compared to placebo (*p* < 0.001 vs. placebo for all groups). In the “with-statin” group, the estimated percent change from baseline was −2.0% for placebo and −45.1%, −48.5%, and −50.0%, respectively, for the pemafibrate groups. Findings for both groups showed significant decreases in TG-rich lipoproteins and atherogenic lipid parameters compared to placebo. The incidence of adverse events was similar between the pemafibrate and placebo groups and was also similar for patients with and without renal dysfunction in the “with-statin” group. Pemafibrate lowered TG and improved atherogenic dyslipidemia without a significant increase in adverse events in comparison to the placebo, even among “with-statin” patients who had renal dysfunction.

## 1. Introduction 

Numerous risk factors impact the development of atherosclerosis and cardiovascular (CV) events, including diabetes mellitus, hypertension, habitual smoking, stress, and the widely recognized factor of dyslipidemia [1]. As first evidenced in the 4S study [2], management of low-density lipoprotein-cholesterol (LDL-C) with statins can be highly effective for CV event prevention, and this approach is currently widely recommended [3,4]. However, lowering LDL-C is not sufficient for such prevention; hypertriglyceridemia has emerged as an independent risk factor, even when LDL-C levels are well controlled by statins [5]. Further add-on therapy is needed to reduce the risk associated with high triglyceride (TG) concentration. The peroxisome proliferator-activated receptor α (PPARα) agonists are potential candidates for this type of add-on therapy [6].

Pemafibrate is a potent selective PPARα modulator (SPPARMα) that has a favorable benefit-risk balance and may be safer than conventional PPARα agonists [7,8]. Pemafibrate regulates human hepatic gene expression differently from existing agents [9], as the unique Y-shaped structure of the pemafibrate molecule efficiently occupies all areas of the Y-shaped PPARα ligand binding site [10]. In clinical use, pemafibrate can effectively lower TG levels while providing a favorable adverse event (AE) profile and reducing the incidence of abnormal liver/renal function tests compared with conventional PPARα agonists [11,12]. Additionally, unlike existing agents, pemafibrate is principally excreted via the liver rather than via the kidneys [13]. Elevated plasma pemafibrate concentrations have not been noted in single-dose or repeated-dose studies of patients with renal dysfunction [14,15], or in drug-drug interaction studies of pemafibrate with various statins [16], suggesting that pemafibrate may represent a new drug category distinct from the conventional PPARα agonists.

These findings have focused attention on the efficacy and safety of pemafibrate in patients treated with statins. However, the efficacy and safety of pemafibrate with and without statin therapy could not be tested in the individual studies because of the small number of patients in each study. Such a study could build on the results from an earlier study showing that concomitant pemafibrate with various statins did not affect statin concentration in the blood [16] and could help determine how such use affects clinical efficacy and safety and whether those findings can be applied to larger groups of patients with hypertriglyceridemia. In the present study, we investigated and compared the efficacy and safety of pemafibrate in detail with and without concomitant statins by using pooled data from six phase 2 and phase 3 placebo-controlled studies of pemafibrate [11,17,18,19,20]. 

In recent years, lipoprotein subclass profiles are increasingly considered as key tools for assessing the effects of lipid-lowering therapies. Gel filtration high-performance liquid chromatography (HPLC) permits the assessment of a greater number of lipid subfractions [21]. Thus, this study includes data detailing the lipid subclasses obtained with HPLC. 

In addition, little information is available on the efficacy and safety of concomitant PPARα agonists and statin therapies in patients with chronic kidney disease. We thus performed additional analysis of the effects of pemafibrate on plasma lipids, and the safety of such use, in patients with renal dysfunction in the group treated with concomitant statins.

## 2. Results

### 2.1. Patient Characteristics

The subjects of the current study consisted of 1253 patients in the full analysis set (FAS) population: 677 in the “with-statin” group and 576 in the “without-statin” group. Within the concomitant statin group, 96 patients had renal dysfunction (baseline estimated glomerular filtration rate (eGFR) < 60 mL/min/1.73 m^2^) and 581 patients had normal renal function or mild renal impairment (baseline eGFR ≥ 60 mL/min/1.73 m^2^). The safety analysis set consisted of 1255 patients: 677 in the “with-statin” group and 578 in the “without-statin” group (Figure S1). 

Patients in the “with-statin” group (mean (standard deviation)) were older (56.6 (11.1) yr vs. 51.6 (11.1) yr, included a higher percentage of female patients (19.8% vs. 8.7%), had a higher prevalence of diabetes (41.4% vs. 29.3%), hypertension (62.2% vs. 31.4%), and fatty liver (56.3% vs. 26.6%), and had lower LDL-C (2.91 (0.75) mmol/L vs. 3.50 (0.95) mmol/L) and higher hemoglobin A1c (HbA1c, 6.43 (0.80)% vs. 6.06 (0.75)%) than patients in the “without-statin” group (Table 1). Patients with renal dysfunction were older and had lower body mass index (BMI) than patients with normal or mildly impaired renal function (Table S1).

### 2.2. Efficacy

#### 2.2.1. Effects on TG

**Figure 1:**
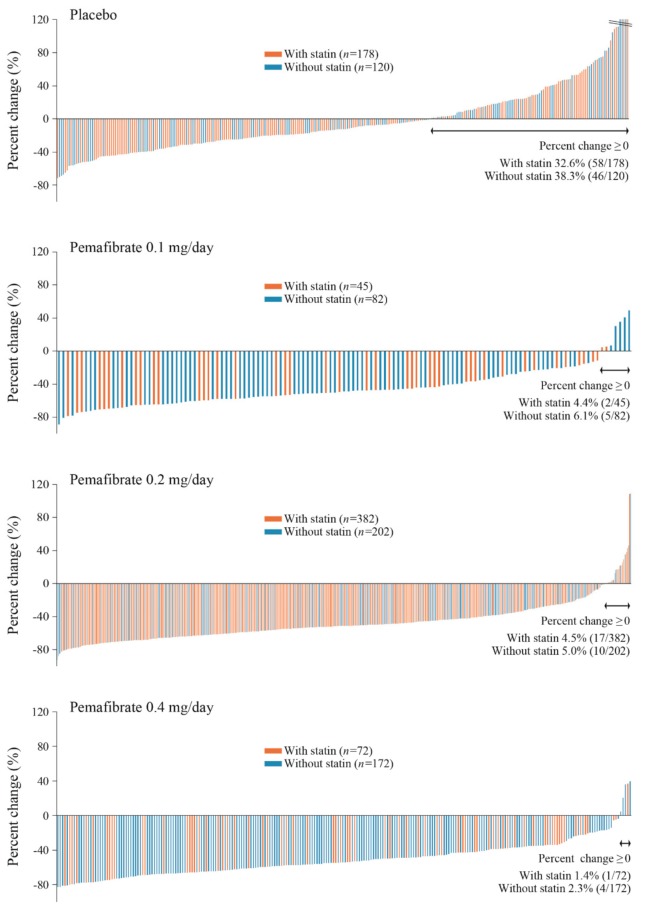
Change in TG from baseline to Week 12. TG, triglyceride.

In both the “with-statin” and the “without-statin” groups, TG was significantly reduced by pemafibrate at doses of 0.1, 0.2, and 0.4 mg/day compared with placebo, with mean percent change in TG as least squares (LS) means (95% confidence interval) ranging from −45.1 (−55.1, −35.1)% for 0.1 mg/day to −50.0 (−57.9, −42.1)% for 0.4 mg/day vs. -2.0 (−7.0, 3.1)% for placebo in the “with-statin” group and from −44.5 (−51.4, −37.6)% for 0.1 mg/day to −51.3 (−56.1, −46.5)% for 0.4 mg/day vs. 1.2 (−4.5, 6.9)% for placebo in the “without-statin” group (*p* < 0.001 for all treatment groups) (Table 2). Based on the distribution of percent change in TG at Week 12 (last observation carried forward [LOCF]), the 0.4 mg/day pemafibrate group contained the lowest percentage of patients who had no reduction in TG (1.4% in the “with-statin” group and 2.3% in the “without-statin” group) (Figure 1).

#### 2.2.2. Effects on HDL-C

**Figure 2:**
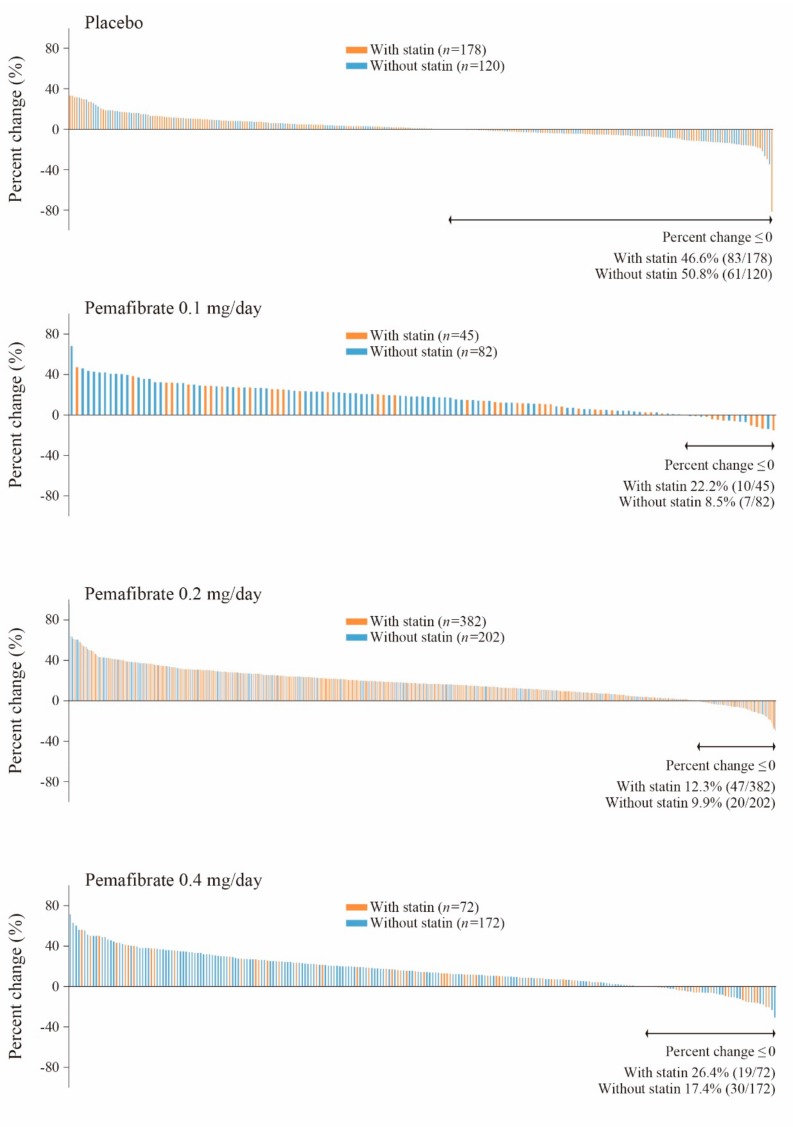
Change in HDL-C from baseline to Week 12. HDL-C, high-density lipoprotein-cholesterol.

In both the “with-statin” and the “without-statin” groups, high-density lipoprotein-cholesterol (HDL-C) increased significantly at all pemafibrate doses compared with placebo. However, the percentage of HDL-C increase was lowest in the pemafibrate 0.4 mg/day group (11.9 (8.5, 15.2)% with statin, 17.8 (15.5, 20.2)% without statin) (Table 2). Findings from HPLC analysis showed a notable decrease in cholesterol concentrations in large HDL (−26.3 (−32.9, −19.8)% with statin, −24.8 (−30.0, −19.5)% without statin) and the greatest increase in small HDL (28.7 (24.8, 32.7)% with statin, 34.5 (31.5, 37.5)% without statin) in the pemafibrate 0.4 mg/day group (Table 3). Based on the distribution of percent change in HDL-C at Week 12 (LOCF), the 0.4 mg/day pemafibrate group contained the highest percentage of patients who had no increase in HDL-C (26.4% with statin, 17.4% without statin) (Figure 2). In that same time period, the 0.4 mg/day group also showed the lowest percentage of patients who had no increase in small HDL-C (10.2%, 5.4%, and 5.4% for the 0.1 mg/day, 0.2 mg/day, and 0.4 mg/day pemafibrate groups, respectively) or very small HDL-C (22.8%, 15.5%, and 12.2% for the 0.1 mg/day, 0.2 mg/day, and 0.4 mg/day pemafibrate groups, respectively).

#### 2.2.3. Effects on LDL-C and Other Lipid Parameters

In both the “with-statin” and the “without-statin” groups, LDL-C increased significantly at pemafibrate 0.2 mg/day (8.8 (6.4, 11.2)% with statin, 11.0 (7.7, 14.4)% without statin, *p* < 0.001 vs. placebo for each) and 0.4 mg/day (7.0 (1.5, 12.5)% with statin, 9.7 (6.0, 13.3)% without statin, *p* < 0.01 vs. placebo for each) compared with placebo (−1.8 (−5.3, 1.7)% with statin, 0.6 (−3.7, 5.0)% without statin) (Table 2). In both the “with-statin” and “without-statin” groups, percent changes in apolipoprotein (Apo) B with pemafibrate treatment did not differ significantly from treatment with placebo, but those values dropped significantly for all groups in comparison to baseline despite the low baseline ApoB value in the “with-statin” group (Table 2). The results of HPLC analysis showed that small LDL-C decreased significantly in the pemafibrate 0.4 mg/day group with statin (−12.2 (−18.2, −6.2)%, *p* < 0.01) and in the 0.2 and 0.4 mg/day groups without statin (-6.8 (−10.6, −3.0)%, *p* < 0.05, and −11.5 (−15.8, −7.1)%, *p* < 0.001, respectively), compared with placebo (−1.2 (−5.0, 2.6)% with statin, 0.0 (−5.1, 5.1)% without statin). Very small LDL-C decreased significantly for all pemafibrate doses, with or without statins, compared with placebo (Table 3). Non-HDL-C decreased significantly for all pemafibrate doses compared with placebo (Table 2). The cholesterol content in TG-rich lipoproteins, such as remnant lipoprotein-cholesterol (RemL-C) (Table 2), chylomicron-cholesterol (CM-C), and very-low-density lipoprotein-cholesterol (VLDL-C) (Table 3), decreased significantly for pemafibrate compared with placebo, regardless of statin use. In both “with-statin” and “without-statin” groups, ApoB48, ApoCIII, ApoCIII/ApoCII ratio, and fibrinogen decreased significantly, and fibroblast growth factor 21 (FGF21) increased significantly, compared with placebo. That improvement was particularly marked for pemafibrate 0.4 mg/day (Table 2).

#### 2.2.4. Analysis Stratified by Presence or Absence of Renal Dysfunction

The effects on lipid parameters, fibrinogen, and FGF21 were investigated in patients receiving statins, with analysis stratified by the presence or absence of renal dysfunction. The results showed similar trends when combining pemafibrate dose groups of 0.1 mg/day to 0.4 mg/day regardless of the presence or absence of renal dysfunction (Tables S2 and S3, Figures S2 and S3).

### 2.3. Safety

#### 2.3.1. Adverse Events and Adverse Drug Reactions 

##### Analysis Stratified by Presence or Absence of Statin 

The total incidence of AEs and of ADRs after 12 weeks was similar to that seen in the placebo groups, regardless of statin use (Table 4). Serious AEs other than death occurred in 15 patients in the pemafibrate groups (eight with statin and seven without statin). The incidence did not increase with concomitant statin therapy. Serious AEs were cervical cancer, upper limb fracture, colon cancer, diabetes mellitus, enterocolitis, hematoma of the abdominal wall, lumbar spinal stenosis, and varicose vein surgery in the “with-statin” groups, and acute myocardial infarction, malignant lung neoplasm, enterocolitis, myocardial infarction, ureteral calculus (in two patients), and bile duct stone in the “without-stain” groups. One patient died in the pemafibrate 0.4 mg/day “without-statin” group due to pulmonary embolism. The death was considered unrelated to the study drug [11].

##### Analysis Stratified by Presence or Absence of Renal Dysfunction in the “with-Statin” Group 

At Week 12, the incidence of AEs and ADRs in the pemafibrate group was similar to that in the placebo group, regardless of the presence or absence of renal dysfunction with statins (Table 5). Serious AEs other than death occurred in five patients with eGFR ≥ 60 mL/min/1.73 m^2^ (cervical cancer, upper limb fracture, colon cancer, diabetes mellitus, enterocolitis) and in three patients with eGFR < 60 mL/min/1.73 m^2^ (hematoma of the abdominal wall, lumbar spinal stenosis, varicose vein surgery). Patients with renal dysfunction showed no increase in the incidence of serious adverse events. No deaths occurred in either “with-statin” group during the study.

#### 2.3.2. Safety Evaluation Using Cutoff Values for AST, ALT, sCr, and CK

##### Analysis Stratified by Presence or Absence of a Statin 

An increase in aspartate aminotransferase (AST) more than three times the upper limit of the normal range (40 U/L) was noted at least once during the study period of 12 weeks in one patient who received pemafibrate without statin and two patients who received pemafibrate with statin (Table 4). During that same time period, an alanine aminotransferase (ALT) increase to more than three times the upper limit of the normal range (45 U/L) was noted at least once in two patients receiving pemafibrate with statin. During the 12-week period, the pemafibrate groups and placebo group showed similar percentages of patients whose creatine kinase (CK) increased to more than 2.5, 5, or 10 times the upper limit of normal range (270 U/L for males and 150 U/L for females) at least once, regardless of statin use. CK increased more than 10 times the upper limit of normal range in two patients, one in the “with-statin group” with eGFR ≥ 60 mL/min/1.73 m^2^ receiving pemafibrate 0.2 mg/day (CK peaked at 3725 U/L in Week 2), and the other in the “without-statin” group with eGFR ≥ 60 mL/min/1.73 m^2^ receiving pemafibrate 0.4 mg/day (CK peaked at 6430 U/L in Week 8). Both patients were discontinued from the study and recovered spontaneously. Neither patient experienced drug-associated muscle symptoms.

##### Analysis Stratified by Presence or Absence of Renal Dysfunction in the “with-Statin” Groups 

During Week 12, AST increased to more than three times the upper limit of the normal range (40 U/L) at least once in one pemafibrate-treated patient with eGFR < 60 mL/min/1.73 m^2^ and in one pemafibrate-treated patient with eGFR ≥ 60 mL/min/1.73 m^2^ (Table 5). ALT increased to more than three times the upper limit of normal range (45 U/L) at least once in two pemafibrate-treated patients with eGFR ≥ 60 mL/min/1.73 m^2^. During the 12-week period, patients with eGFR < 60 mL/min/1.73 m^2^ were more likely to show serum creatinine (sCr) increased to more than the upper limit of normal range (1.04 mg/dL for males and 0.79 mg/dL for females) at least once. The same trend was seen in the placebo groups. The incidence of sCr increasing to more than the upper limit of normal was similar for placebo and all pemafibrate doses. The pemafibrate groups and placebo group showed similar percentages of patients whose CK increased to more than 2.5, 5, or 10 times the upper limit of normal range (270 U/L for males and 150 U/L for females) at least once, regardless of presence or absence of renal dysfunction. No patients with eGFR < 60 mL/min/1.73 m^2^ showed CK increases of more than 10 times the upper limit of normal range.

#### 2.3.3. Renal Function, CK, and Liver Function 

##### Analysis by Presence or Absence of Statin 

Compared with placebo, sCr increased significantly in groups receiving pemafibrate 0.2 mg/day or above (least mean square, + 0.02 mg/dL for 0.2 mg/day and ranging from + 0.04 to + 0.05 mg/dL for 0.4 mg/day), regardless of statin use (Table 6). The eGFR findings were also significantly reduced in groups treated with pemafibrate 0.2 mg/day or above, regardless of statin use. No notable fluctuations were seen in mean CK values. ALT and gamma-glutamyltransferase (γ-GT) decreased more in the pemafibrate dose groups than placebo groups, regardless of statin use.

##### Analysis by Presence or Absence of Renal Dysfunction in “with-Statin” Groups 

In patients with eGFR ≥60 mL/min/1.73 m^2^, sCr increased significantly (0.03 mg/dL vs. 0.00 mg/dL) and eGFR decreased significantly (−2.8 vs. −0.2 mL/min/1.73 m^2^) for pemafibrate 0.1–0.4 mg/day compared with placebo (Table S4). In renal dysfunction patients with eGFR < 60 mL/min/1.73 m^2^, no significant differences were noted in changes in serum creatinine or eGFR between placebo and pemafibrate.

## 3. Discussion

This study examined combined data from six randomized controlled trials to determine the efficacy and safety of using pemafibrate with statins. A TG decrease of approximately 50% was noted in all groups treated with pemafibrate, regardless of statin use or renal dysfunction. This trend was consistent with the previous findings obtained in each individual study [11,17,18,19,20]. The percentage of patients who experienced no decrease in TG (i.e., non-responders) was lowest in the pemafibrate 0.4 mg/day group. 

Significant decreases in large HDL-C were seen in the groups receiving pemafibrate 0.4 mg/day, regardless of statin use. Dose-dependent increases were noted in small HDL-C, which is considered more functional and thus more atheroprotective. Our study of pemafibrate to assess the effects on HDL function showed that cholesterol efflux capacity (CEC) was improved with administration of pemafibrate 0.4 mg/day compared to placebo [22]. This is of interest because CEC is inversely correlated with cardiac risk and may be used to estimate HDL function and the activities of adenosine triphosphate (ATP)-binding cassette transporter A1 (ABCA1) and ATP-binding cassette transporter G1 (ABCG1). Our findings are consistent with other research that pemafibrate may enhance the expression of ABCA1 and ABCG1 [23]. The decrease in small particle LDL-C, which is well-known to be atherogenic, was greatest for pemafibrate 0.4 mg/day. 

Decreases in ApoB48, ApoCIII, ApoCIII/ApoCII, and fibrinogen were greatest in the 0.4 mg/day group. A decrease in ApoB48 reflects the capability of pemafibrate to inhibit the absorption of exogenous cholesterol [24,25]. Findings from basic research suggest that pemafibrate may also inhibit expression of Niemann-Pick C 1-Like-1 (NPC1L1) mRNA [26,27]. Decreases in ApoCIII and ApoCIII/ApoCII may reflect accelerated catabolism of TG by lipoprotein lipase (LPL) [28]. Basic data are available on the mechanism by which pemafibrate enhances LPL activity [26]. Fibrinogen also contributes to vascular inflammation and atherosclerosis [29]. Other basic data indicate that pemafibrate decreases the expression of vascular cell adhesion molecule-1 (VCAM-1), macrophage marker F4/80, and interleukin (IL)-6 in a dose-dependent manner [23]. All these findings collectively indicate that 0.4 mg/day of pemafibrate may improve atherogenic dyslipidemia. Further studies, including the Pemafibrate to Reduce Cardiovascular OutcoMes by Reducing Triglycerides IN patiENts with diabeTes (PROMINENT) study for prevention of CV events, are ongoing to evaluate 0.4 mg/day administration of pemafibrate [30]. 

In this study, we found that renal dysfunction did not affect the efficacy of pemafibrate-statin combinations. We previously reported the effects of 52 weeks of pemafibrate treatment in patients with various levels of renal dysfunction. In that study, decreased renal function was associated with lower HDL-C values at baseline, and a notable increase in HDL-C and decrease in small-particle LDL-C during pemafibrate treatment were found at the lowest level of renal function (eGFR < 30 mL/min/1.73 m^2^) [15]. In this pooled analysis, the population included only one patient with eGFR < 30 mL/min/1.73 m^2^. We were thus unable to follow up those findings.

Findings from our pooled analyses showed no increase in AEs compared with placebo in “with-statin” or “without-statin” groups, and no increase in AEs among renal dysfunction patients in the “with-statin” group compared with placebo. Other studies found that increases in plasma concentration were not related to increased renal dysfunction following single or repeated administration of pemafibrate in patients with impaired renal function, possibly because pemafibrate is excreted primarily through the liver and feces [13], and a drug-drug interaction study with concomitant statin showed no increase in plasma concentration of pemafibrate or statin [16]. The safety results from our study support those findings.

The combined use of PPARα agonists and statins is a useful option for the treatment of atherogenic dyslipidemia [6]. However, the combination of cerivastatin and gemfibrozil has been associated with a 559% increase in the plasma concentration of cerivastatin [31]. In addition, according to data from the Adverse Event Reporting System of the Food and Drug Administration, rhabdomyolysis increased specifically in this combination [32,33]. Although these findings raise the level of caution required for the use of statin-PPARα agonist combinations, statin-PPARα agonist studies including the ACCORD-LIPID trial using fenofibrate and simvastatin [34] and the FIRST trial using fenofibric acid and atorvastatin [35] have shown no increase of rhabdomyolysis. However, because most PPARα agonists are excreted primarily through the kidneys, patients with renal dysfunction may experience cumulative renal toxicity requiring dose adjustment, which is a limitation of this treatment regimen [36].

In the context of safety cut-off values, no marked fluctuation was seen in CK regardless of statin use, and liver enzyme behaviors were similar to previous findings. Only four patients showed AST or ALT increases more than three times the upper limit of the normal range. Neither of the two patients whose CK increased more than 10 times the upper limit of normal range had renal dysfunction. One was a 38-year-old man whose CK reached 3725 U/L with no complaint of muscle symptoms after administration for two weeks. The condition resolved without additional interventions 22 days after the incident. A causal relationship to the study drug could not be ruled out by the investigators [18]. The other patient also recovered without incident. In that case also, a causal relationship to the study drug could not be ruled out.

Regardless of statin use, no marked fluctuation was seen in CK, and liver enzymes showed behaviors that were similar to previous findings. In contrast to the elevation of ALT and γ-GT that was seen with fenofibrate [11,12], both ALT and γ-GT were reduced with pemafibrate. The level of sCr increased very slightly, and eGFR decreased slightly, in a dose-dependent manner regardless of statin use. In the pemafibrate 0.4 mg/day group, the LS mean change in sCr was 0.04 mg/dL without statin and 0.05 mg/dL with statin. These values were markedly less than in patients treated with high-dose fenofibrate, who experienced sCr increases of 0.1 mg/dL to 0.2 mg/dL [11,34]. These findings suggest that the PPARα modulation seen with pemafibrate causes less increase in sCr than is seen with the standard PPARα agonists.

There were no noteworthy findings for adverse events, adverse drug reactions, or cutoffs for pemafibrate with a concomitant statin in patients with renal dysfunction, and there was no increase in CK or sCr. These findings suggested that pemafibrate can be safely used with a concomitant statin even in this patient population.

The metabolic profile of pemafibrate means that the drug is safe for patients with renal dysfunction. In addition, no clinically meaningful elevation of plasma statin has been reported in patients with renal dysfunction [37,38,39,40]. As noted above, we found no notable drug-drug interactions in our studies. These findings indicate that the combined use of pemafibrate and statins provides a promising treatment option for patients with renal dysfunction, who are particularly prone to accumulation of lipoprotein remnants and reduction of HDL-C [41,42]. 

LDL-C lowering therapy with statins is known to effectively reduce CV events in early-stage chronic kidney disease (CKD) [43], but not in patients under dialysis, as shown in the 4D and AURORA studies [44,45]. As of this writing, only ezetimibe, which selectively inhibits the absorption of cholesterol from the small intestine, has been found to reliably reduce CV events in patients with CKD, including those on dialysis in the SHARP trial (in combination with simvastatin) [46]. In the future, we anticipate increased interest in the clinical efficacy of pemafibrate in patients with renal dysfunction who are at very high risk of CV events [47,48]. 

This study has several limitations. It included only Japanese subjects, which means that racial differences remain to be investigated. Almost no patients with severe renal dysfunction (eGFR < 30 mL/min/1.73 m^2^) were included, so we can draw no conclusions about that patient population. In addition, no data were available from clinical follow-up for the reduction of CV events. We hope that the PROMINENT study [30], currently underway, will provide more information in this area. Furthermore, the use of pooled analyses could have introduced inter-group bias at baseline for some parameters. Finally, more long-term safety data are needed. 

## 4. Subjects and Methods

The present study analyzed data combined from six randomized double-blind placebo-controlled studies that were conducted in Japan (Table S5) and continued for 12 weeks unless otherwise noted: a phase 2 study in 224 patients with a history of documented dyslipidemia and fasting plasma TG of 2.26 mmol/L (200 mg/dL) or higher, randomized to placebo, pemafibrate 0.05, 0.1, 0.2, or 0.4 mg/day, or fenofibrate 100 mg/day [17]; a phase 3 study in 526 patients with dyslipidemia, high fasting TG levels, and low HDL-C levels, randomized to placebo, pemafibrate 0.1, 0.2, or 0.4 mg/day, or fenofibrate 100 or 200 mg/day [11]; a study in 188 patients with dyslipidemia who were placed under treatment with pitavastatin at a starting dose of 2 mg, and after at least four weeks of this treatment were then randomized to placebo, pemafibrate 0.1, 0.2, or 0.4 mg/day in combination with pitavastatin [18]; a 24-week study in 423 patients with dyslipidemia, randomized to placebo, pemafibrate 0.2 mg/day (fixed dose) or 0.2 (0.4) mg/day (conditional up-titration) with any statin [18]; a 24-week phase 3 study in 166 patients with type 2 diabetes and hypertriglyceridemia, randomized to placebo, pemafibrate 0.2 or 0.4 mg/day [19]; a study in 27 patients with hypertriglyceridemia and insulin resistance, randomized to pemafibrate 0.4 mg/day or placebo [20]. All studies were approved by the Institutional Review Board for each study institution and were conducted in accordance with the Declaration of Helsinki and under the guidelines of Good Clinical Practice. All study patients provided written informed consent prior to enrollment in each study. All studies were supported by Kowa Company, Ltd. This pooled analysis was approved by the Ethics Committee of the Rinku General Medical Center (Application No. 2019-022), with which the first author of this paper is affiliated.

In this study, we evaluated the efficacy and safety of pemafibrate 0.1 mg/day, 0.2 mg/day, and 0.4 mg/day (twice daily), with and without a statin. The primary efficacy endpoint was the percent change in TG from baseline to 12 weeks. The secondary efficacy endpoints were also assessed from baseline to 12 weeks: percent change in HDL-C, LDL-C, non-HDL-C, total cholesterol (TC), RemL-C, ApoAI, ApoAII, ApoB, ApoB48, ApoB100, ApoCII, ApoCIII, ApoCIII/ApoCII, and ApoE; percent change in HPLC findings for CM-C, VLDL-C, cholesterol content in four subclasses of LDL (large, medium, small, and very small LDLs), and cholesterol content for five subclasses of HDL (very large, large, medium, small, and very small HDLs); and changes in fibrinogen and FGF21. The primary safety endpoints were the incidence of adverse events and adverse drug reactions. Secondary safety endpoints were percentage of values above the upper limit of normal range for AST, ALT, sCr, and CK, and change in sCr, eGFR, CK, AST, ALT, γ-GT, alkaline phosphatase (ALP), and total bilirubin. All hematological data are from fasting blood samples (drawn at least 10 hours after the patient’s most recent meal).

Patients with renal dysfunction were defined as those with baseline eGFR < 60 mL/min/1.73 m^2^ (eGFR_male_ = 194 × sCr ^−1.094^ × age^−0.287^, eGFR_female_ = 194 × sCr^−1.094^ ×age^−0.287^ × 0.739 [49]). The analysis was stratified by the presence or absence of renal dysfunction in the concomitant statin group, and efficacy and safety of pemafibrate were evaluated. In the efficacy and safety evaluations excluding AEs, ADRs, and cutoff values for AST, ALT, sCr, and CK, findings were combined for pemafibrate subgroups receiving doses of 0.1 mg/day, 0.2 mg/day, and 0.4 mg/day.

For each lipid parameter, gel filtration HPLC was performed at Skylight Biotech, Inc. Other measurements were performed at LSI Medience Corporation. The FAS analysis set was used for efficacy parameters regarding effects on lipids, fibrinogen, and FGF21. The FAS was defined as all subjects who were randomized and took at least one dose of placebo or pemafibrate, and for whom baseline and at least one post-baseline value was available for assessment of efficacy endpoints. The safety analysis set was used for safety parameters and was defined as all subjects who were randomized and took at least one dose of placebo or pemafibrate. For lipid parameters, fibrinogen, and FGF21, LS means (95% confidence interval) were calculated at Week 12 (LOCF) using analysis of covariance (ANCOVA) with baseline values as the co-variable within each category. For safety parameters, LS means (95% confidence interval) was calculated at Week 12 using ANCOVA with baseline values as the co-variable within each category. Multiplicity was not considered in any of the statistical analyses in this study. SAS version 9.2 was used for analyses.

## 5. Conclusions

We found that 12 weeks of treatment with pemafibrate lowered TG and contributed to the overall improvement of atherogenic dyslipidemia with no increase in the incidence of adverse events compared with placebo, regardless of statin use. Similar results were observed for concomitant statin use in patients with renal dysfunction. Pemafibrate seems promising for improving lipid profiles in a variety of populations with dyslipidemia.

## Figures and Tables

**Table 1:** Characteristics of patients at baseline (FAS).

Parameter	With Statin					Without Statin				
	Placebo		Pemafibrate		All	Placebo		Pemafibrate		All
		0.1 mg/day	0.2 mg/day	0.4 mg/day			0.1 mg/day	0.2 mg/day	0.4 mg/day	
*n*	178	45	382	72	677	120	82	202	172	576
Age (years)	56.9 (11.4)	55.0 (10.3)	56.9 (11.1)	55.7 (11.1)	56.6 (11.1)	52.0 (10.6)	50.0 (11.3)	52.0 (11.4)	51.6 (11.1)	51.6 (11.1)
Age ≥ 65 years	44 (24.7)	7 (15.6)	103 (27.0)	13 (18.1)	167 (24.7)	18 (15.0)	9 (11.0)	27 (13.4)	27 (15.7)	81 (14.1)
Sex, Female	38 (21.3)	9 (20.0)	73 (19.1)	14 (19.4)	134 (19.8)	15 (12.5)	2 (2.4)	21 (10.4)	12 (7.0)	50 (8.7)
Body weight (kg)	74.73 (14.05)	75.76 (12.58)	74.35 (14.14) ^1^	73.03 (13.05)	74.40 (13.89) ^2^	75.75 (12.45)	77.15 (11.74)	76.82 (13.43)	74.88 (12.20)	76.07 (12.64)
BMI (kg/m^2^)	27.22 (3.71)	27.38 (3.70)	27.18 (4.01) ^1^	26.35 (3.43)	27.12 (3.85) ^2^	26.45 (3.41)	26.88 (3.57)	26.87 (3.60)	26.21 (3.46)	26.59 (3.52)
BMI ≥ 25 kg/m^2^	125 (70.2)	34 (75.6)	257 (67.3)	43 (59.7)	459 (67.8)	75 (62.5)	53 (64.6)	131 (64.9)	102 (59.3)	361 (62.7)
Type 2 diabetes	72 (40.4)	15 (33.3)	154 (40.3)	39 (54.2)	280 (41.4)	44 (36.7)	8 (9.8)	65 (32.2)	52 (30.2)	169 (29.3)
Hypertension	107 (60.1)	20 (44.4)	251 (65.7)	43 (59.7)	421 (62.2)	42 (35.0)	23 (28.0)	71 (35.1)	45 (26.2)	181 (31.4)
Fatty liver	106 (59.6)	13 (28.9)	235 (61.5)	27 (37.5)	381 (56.3)	37 (30.8)	15 (18.3)	60 (29.7)	41 (23.8)	153 (26.6)
Pravastatin	22 (12.4)	0	49 (12.8)	0	71 (10.5)	0	0	0	0	0
Simvastatin	5 (2.8)	0	7 (1.8)	1 (1.4)	13 (1.9)	0	0	0	0	0
Fluvastatin	4 (2.2)	0	8 (2.1)	4 (5.6)	16 (2.4)	0	0	0	0	0
Atorvastatin	30 (16.9)	0	76 (19.9) ^3^	3 (4.2)	109 (16.1)	0	0	0	0	0
Pitavastatin	73 (41.0)	45 (100)	134 (35.1)	57 (79.2)	309 (45.6)	0	0	0	0	0
Rosuvastatin	44 (24.7) ^4^	0	108 (28.3) ^3^	7 (9.7)	159 (23.5)	0	0	0	0	0
TG (mmol/L)	3.78 (1.72)	3.88 (1.43)	3.69 (1.52)	3.62 (1.35)	3.72 (1.55)	3.69 (1.36)	3.71 (1.08)	3.80 (1.60)	3.75 (1.61)	3.75 (1.49)
HDL-C (mmol/L)	1.18 (0.27)	1.25 (0.22)	1.19 (0.26)	1.19 (0.22)	1.19 (0.26)	1.07 (0.19)	1.01 (0.14)	1.04 (0.19)	1.07 (0.44)	1.05 (0.28)
LDL-C (mmol/L)	2.89 (0.76)	3.24 (0.51)	2.85 (0.78)	3.10 (0.61)	2.91 (0.75)	3.61 (0.87)	3.39 (0.96)	3.49 (0.96)	3.49 (0.97)	3.50 (0.95)
HbA1c (%)	6.39 (0.76)	6.31 (0.61)	6.42 (0.85)	6.60 (0.65)	6.43 (0.80)	6.17 (0.78)	5.69 (0.59)	6.11 (0.74)	6.09 (0.76)	6.06 (0.75)
eGFR ^5^ (mL/min/1.73 m^2^)	76.4 (18.5)	78.2 (17.6)	77.8 (16.7)	78.1 (17.0)	77.5 (17.3)	78.7 (15.4)	75.9 (14.5)	79.2 (15.7)	78.9 (15.3)	78.5 (15.3)

Data are presented as mean (standard deviation) for continuous parameters and the number of patients (percentage) for categorical parameters. ^1^ *n* = 381, ^2^ *n* = 676, ^3^ one patient switched from atorvastatin to rosuvastatin at week 2. ^4^ including one patient who started rosuvastatin at week 8. ^5^ eGFR_male_ = 194 × sCr^−1.094^ × age^−0.287^, eGFR_female_ = 194 × sCr^−1.094^ × age^−0.287^ × 0.739. FAS, full analysis set; BMI, body mass index; TG, triglyceride; HDL-C, high-density lipoprotein-cholesterol; LDL-C, low-density lipoprotein-cholesterol; HbA1c, hemoglobin A1c; eGFR, estimated glomerular filtration rate; sCr, serum creatinine.

**Table 2:** Changes in lipoproteins, fibrinogen, and FGF21 from baseline to Week 12 (FAS).

**(A) With Statin**					
**Parameter**		**Placebo**		**Pemafibrate**	
			**0.1 mg/day**	**0.2 mg/day**	**0.4 mg/day**
TG (mmol/L)	*n*	178	45	382	72
	Baseline	3.78 (1.72)	3.88 (1.43)	3.69 (1.52)	3.62 (1.35)
	Week 12 (LOCF)	3.74 (4.73)	2.12 (1.22)	1.83 (0.97)	1.72 (0.87)
	% Change	−2.0 (−7.0, 3.1)	−45.1 (−55.1, −35.1) ***	−48.5 (−51.9, −45.1) ***	−50.0 (−57.9, −42.1) ***
HDL-C (mmol/L)	*n*	178	45	382	72
	Baseline	1.18 (0.27)	1.25 (0.22)	1.19 (0.26)	1.19 (0.22)
	Week 12 (LOCF)	1.20 (0.29)	1.41 (0.33)	1.38 (0.33)	1.31 (0.25)
	% Change	1.7 (−0.4, 3.9)	13.2 (8.9, 17.4) ***	16.5 (15.1, 18.0) ***	11.9 (8.5, 15.2) ***
LDL-C (mmol/L)	*n*	178	45	382	72
	Baseline	2.89 (0.76)	3.24 (0.51)	2.85 (0.78)	3.10 (0.61)
	Week 12 (LOCF)	2.82 (0.79)	3.22 (0.83)	2.99 (0.72)	3.17 (0.85)
	% Change	−1.8 (−5.3, 1.7)	5.2 (−1.8, 12.1)	8.8 (6.4, 11.2) ***	7.0 (1.5, 12.5) **
Non-HDL-C (mmol/L)	*n*	178	45	382	72
	Baseline	4.05 (0.70)	4.43 (0.56)	3.99 (0.74)	4.24 (0.68)
	Week 12 (LOCF)	3.94 (1.34)	3.92 (0.95)	3.62 (0.77)	3.83 (0.96)
	% Change	−2.5 (−5.3, 0.3)	−9.7 (−15.3, −4.1) *	−8.7 (−10.6, −6.8) ***	−8.0 (−12.4, −3.5) *
TC (mmol/L)	*n*	178	45	382	72
	Baseline	5.23 (0.77)	5.68 (0.60)	5.18 (0.79)	5.43 (0.69)
	Week 12 (LOCF)	5.14 (1.31)	5.33 (0.99)	5.00 (0.75)	5.14 (0.89)
	% Change	−1.6 (−3.7, 0.5)	−3.8 (−8.1, 0.4)	−3.1 (−4.5, −1.6)	−3.6 (−6.9, −0.3)
RemL-C (mmol/L)	*n*	44	44	49	47
	Baseline	0.66 (0.38)	0.61 (0.36)	0.64 (0.45)	0.69 (0.44)
	Week 12 (LOCF)	0.62 (0.31)	0.32 (0.25)	0.26 (0.17)	0.28 (0.23)
	% Change	13.9 (0.8, 26.9)	−42.5 (−55.6, −29.5) ***	−49.2 (−61.5, −36.8) ***	−50.0 (−62.6, −37.3) ***
ApoAI (mg/dL)	*n*	148	44	361	47
	Baseline	138.4 (21.9)	142.4 (16.9)	136.6 (21.1)	136.2 (16.9)
	Week 12 (LOCF)	137.0 (22.1)	146.0 (21.3)	142.7 (19.5)	137.5 (16.2)
	% Change	−0.6 (−2.0, 0.7)	3.2 (0.7, 5.8) **	4.9 (4.1, 5.8) ***	1.6 (−0.9, 4.0)
**(A) With Statin**					
**Parameter**		**Placebo**		**Pemafibrate**	
			**0.1 mg/day**	**0.2 mg/day**	**0.4 mg/day**
ApoAII (mg/dL)	*n*	148	44	361	47
	Baseline	32.0 (4.8)	33.7 (4.6)	31.8 (4.9)	32.6 (4.4)
	Week 12 (LOCF)	31.9 (5.1)	39.9 (8.5)	40.1 (7.2)	41.8 (7.2)
	% Change	−0.3 (−2.7, 2.2)	18.9 (14.4, 23.4) ***	26.8 (25.3, 28.4) ***	29.1 (24.8, 33.5) ***
ApoB (mg/dL)	*n*	148	44	361	47
	Baseline	98.7 (17.9)	105.8 (13.0)	96.1 (19.0)	103.6 (12.9)
	Week 12 (LOCF)	94.0 (16.6)	96.9 (21.1)	89.8 (18.7)	94.7 (17.9)
	% Change	−3.7 (−6.2, −1.2)	−5.8 (−10.5, −1.2)	−5.8 (−7.5, −4.2)	−5.9 (−10.3, −1.4)
ApoB48 (μg/mL)	*n*	46	45	49	47
	Baseline	13.9 (10.2)	11.0 (7.0)	12.5 (13.8)	12.1 (7.7)
	Week 12 (LOCF)	14.2 (15.3)	5.7 (4.5)	4.3 (3.2)	4.5 (4.0)
	% Change	27.3 (10.7, 43.9)	−45.5 (−62.3, −28.7) ***	−55.1 (−71.2, −39.0) ***	−59.7 (−76.1, −43.3) ***
ApoB100 (mg/dL)	n	44	44	49	47
	Baseline	102.7 (17.6)	104.7 (12.8)	104.5 (18.8)	102.4 (12.8)
	Week 12 (LOCF)	98.7 (16.9)	96.3 (20.9)	94.9 (18.9)	94.2 (17.7)
	% Change	−3.6 (−8.2, 1.0)	−7.7 (−12.3, −3.2)	−7.4 (−11.8, −3.1)	−7.5 (−11.9, −3.0)
ApoCII (mg/dL)	*n*	148	44	361	47
	Baseline	8.1 (2.6)	8.2 (3.0)	8.2 (2.5)	8.6 (2.1)
	Week 12 (LOCF)	7.9 (2.2)	6.7 (2.4)	6.4 (2.2)	6.4 (2.5)
	% Change	−0.2 (−3.7, 3.3)	−16.0 (−22.3, −9.6) ***	−20.6 (−22.8, −18.4) ***	−24.5 (−30.7, −18.4) ***
ApoCIII (mg/dL)	*n*	148	44	361	47
	Baseline	17.7 (6.4)	17.5 (6.7)	17.1 (5.6)	18.3 (5.2)
	Week 12 (LOCF)	16.9 (5.8)	12.8 (5.6)	11.1 (3.9)	10.6 (3.7)
	% Change	−0.3 (−3.6, 3.0)	−25.0 (−31.0, −18.9) ***	−33.2 (−35.3, −31.1) ***	−38.1 (−44.0, −32.3) ***
ApoCIII/ApoCII	*n*	148	44	361	47
	Baseline	2.2 (0.5)	2.2 (0.5)	2.1 (0.5)	2.2 (0.5)
	Week 12 (LOCF)	2.2 (0.5)	2.0 (0.5)	1.8 (0.4)	1.7 (0.4)
	% Change	0.6 (−2.0, 3.2)	−8.5 (−13.3, −3.7) **	−14.6 (−16.3, −12.9) ***	−17.9 (−22.6, −13.3) ***
**(A) With Statin**					
**Parameter**		**Placebo**		**Pemafibrate**	
			**0.1 mg/day**	**0.2 mg/day**	**0.4 mg/day**
ApoE (mg/dL)	*n*	148	44	361	47
	Baseline	5.4 (2.0)	5.3 (2.2)	5.2 (1.7)	5.5 (2.1)
	Week 12 (LOCF)	5.2 (1.6)	4.2 (1.2)	4.0 (0.9)	4.0 (1.1)
	% Change	1.0 (−2.1, 4.1)	−13.7 (−19.4, −8.1) ***	−19.6 (−21.6, −17.6) ***	−20.3 (−25.8, −14.9) ***
Fibrinogen (mg/dL)	*n*	177	45	381	72
	Baseline	285.8 (49.0)	278.6 (48.7)	287.8 (54.1)	285.3 (46.9)
	Week 12 (LOCF)	288.8 (50.1)	251.6 (51.1)	242.1 (54.8)	229.2 (43.0)
	Change	2.8 (−3.7, 9.2)	−30.5 (−43.4, −17.7) ***	−45.1 (−49.5, −40.7) ***	−56.6 (−66.8, −46.5) ***
FGF21 (pg/mL)	*n*	46	45	49	47
	Baseline	601.7 (875.8)	429.8 (160.8)	411.4 (182.7)	692.3 (1482.8)
	Week 12 (LOCF)	478.4 (262.3)	718.2 (498.0)	743.0 (402.0)	1156.0 (2107.2)
	Change	−125.5 (−317.2, 66.1)	291.6 (97.7, 485.5) **	335.4 (149.4, 521.4) ***	458.7 (268.4, 648.9) ***
**(B) Without Statin**					
**Parameter**		**Placebo**		**Pemafibrate**	
			**0.1 mg/day**	**0.2 mg/day**	**0.4 mg/day**
TG (mmol/L)	*n*	120	82	202	172
	Baseline	3.69 (1.36)	3.71 (1.08)	3.80 (1.60)	3.75 (1.61)
	Week 12 (LOCF)	3.72 (2.62)	2.01 (1.02)	1.94 (1.00)	1.72 (0.81)
	% Change	1.2 (−4.5, 6.9)	−44.5 (−51.4, −37.6) ***	−45.5 (−49.9, −41.1) ***	−51.3 (−56.1, −46.5) ***
HDL−C (mmol/L)	*n*	120	82	202	172
	Baseline	1.07 (0.19)	1.01 (0.14)	1.04 (0.19)	1.07 (0.44)
	Week 12 (LOCF)	1.08 (0.24)	1.21 (0.23)	1.24 (0.25)	1.24 (0.34)
	% Change	0.8 (−2.0, 3.6)	19.1 (15.7, 22.5) ***	19.7 (17.5, 21.9) ***	17.8 (15.5, 20.2) ***
LDL−C (mmol/L)	*n*	120	82	202	172
	Baseline	3.61 (0.87)	3.39 (0.96)	3.49 (0.96)	3.49 (0.97)
	Week 12 (LOCF)	3.52 (0.86)	3.48 (0.88)	3.71 (0.95)	3.66 (0.85)
	% Change	0.6 (−3.7, 5.0)	5.0 (−0.3, 10.3)	11.0 (7.7, 14.4) ***	9.7 (6.0, 13.3) **
Non-HDL-C (mmol/L)	*n*	120	82	202	172
	Baseline	4.80 (0.79)	4.57 (0.92)	4.74 (0.95)	4.67 (0.96)
	Week 12 (LOCF)	4.71 (0.81)	4.15 (0.92)	4.39 (1.08)	4.31 (0.91)
	% Change	−1.0 (−3.6, 1.5)	−8.7 (−11.9, −5.6) ***	−6.8 (−8.8, −4.8) ***	−6.6 (−8.8, −4.5) **
**(B) Without Statin**					
**Parameter**		**Placebo**		**Pemafibrate**	
			**0.1 mg/day**	**0.2 mg/day**	**0.4 mg/day**
TC (mmol/L)	*n*	120	82	202	172
	Baseline	5.88 (0.83)	5.59 (0.97)	5.79 (0.98)	5.75 (1.02)
	Week 12 (LOCF)	5.79 (0.82)	5.37 (0.92)	5.63 (1.07)	5.55 (0.91)
	% Change	−0.7 (−2.7, 1.4)	−4.0 (−6.5, −1.5) *	−2.1 (−3.7, −0.5)	−2.3 (−4.0, −0.6)
RemL-C (mmol/L)	*n*	86	82	159	139
	Baseline	0.59 (0.30)	0.58 (0.30)	0.66 (0.43)	0.65 (0.45)
	Week 12 (LOCF)	0.67 (0.39)	0.30 (0.19)	0.30 (0.20)	0.26 (0.15)
	% Change	24.3 (15.8, 32.9)	−46.6 (−55.4, −37.9) ***	−46.2 (−52.4, −39.9) ***	−48.6 (−55.3, −41.9) ***
ApoAI (mg/dL)	*n*	86	82	161	139
	Baseline	125.7 (12.2)	126.1 (10.7)	127.6 (13.3)	124.8 (12.4)
	Week 12 (LOCF)	125.3 (11.4)	133.5 (14.0)	136.8 (15.5)	134.8 (16.0)
	% Change	0.0 (−2.0, 1.9)	6.0 (4.0, 8.0) ***	7.8 (6.4, 9.3) ***	8.1 (6.5, 9.6) ***
ApoAII (mg/dL)	*n*	86	82	161	139
	Baseline	29.8 (2.9)	29.4 (3.7)	30.3 (3.8)	29.4 (3.5)
	Week 12 (LOCF)	29.4 (3.0)	33.8 (4.5)	36.6 (5.1)	38.3 (5.5)
	% Change	−1.2 (−4.2, 1.7)	14.9 (11.8, 17.9) ***	22.2 (20.0, 24.3) ***	30.3 (28.0, 32.6) ***
ApoB (mg/dL)	*n*	86	82	161	139
	Baseline	113.2 (19.2)	107.7 (20.1)	113.0 (22.6)	109.5 (22.7)
	Week 12 (LOCF)	111.2 (18.4)	102.6 (21.4)	109.9 (25.7)	107.3 (21.3)
	% Change	−0.6 (−3.7, 2.5)	−4.7 (−7.9, −1.5)	−1.3 (−3.6, 0.9)	−0.9 (−3.4, 1.5)
ApoB48 (μg/mL)	*n*	87	82	164	140
	Baseline	10.1 (6.3)	10.5 (6.1)	11.7 (8.7)	11.6 (8.8)
	Week 12 (LOCF)	10.9 (6.8)	4.9 (3.7)	4.5 (3.4)	4.0 (2.7)
	% Change	31.0 (18.2, 43.9)	−46.4 (−59.7, −33.2) ***	−51.1 (−60.4, −41.7) ***	−56.1 (−66.2, −46.0) ***
ApoB100 (mg/dL)	*n*	86	82	161	139
	Baseline	112.2 (19.3)	106.7 (20.1)	111.8 (22.7)	108.3 (22.8)
	Week 12 (LOCF)	110.2 (18.4)	102.1 (21.4)	109.5 (25.6)	106.9 (21.3)
	% Change	−0.6 (−3.7, 2.6)	−4.2 (−7.4, −1.0)	−0.6 (−2.9, 1.7)	−0.2 (−2.7, 2.3)
**(B) Without Statin**					
**Parameter**		**Placebo**		**Pemafibrate**	
			**0.1 mg/day**	**0.2 mg/day**	**0.4 mg/day**
ApoCII (mg/dL)	*n*	86	82	161	139
	Baseline	7.7 (2.4)	7.2 (1.8)	8.3 (3.2)	8.1 (3.3)
	Week 12 (LOCF)	7.9 (2.5)	6.1 (1.8)	6.5 (2.5)	6.2 (2.2)
	% Change	5.0 (0.3, 9.8)	−17.2 (−22.1, −12.3) ***	−17.4 (−20.9, −13.9) ***	−19.7 (−23.4, −15.9) ***
ApoCIII (mg/dL)	*n*	86	82	161	139
	Baseline	15.3 (4.7)	15.2 (4.4)	16.8 (6.2)	15.6 (6.1)
	Week 12 (LOCF)	16.1 (6.0)	10.9 (3.5)	10.8 (3.7)	9.5 (3.1)
	% Change	7.3 (2.5, 12.2)	−26.9 (−31.9, −21.9) ***	−30.1 (−33.7, −26.6) ***	−35.4 (−39.2, −31.6) ***
ApoCIII/ApoCII	*n*	86	82	161	139
	Baseline	2.0 (0.3)	2.1 (0.5)	2.1 (0.6)	2.0 (0.4)
	Week 12 (LOCF)	2.1 (0.5)	1.8 (0.4)	1.7 (0.5)	1.6 (0.4)
	% Change	2.7 (−1.1, 6.6)	−10.4 (−14.3, −6.5) ***	−14.9 (−17.7, −12.1) ***	−17.9 (−20.9, −14.9) ***
ApoE (mg/dL)	*n*	86	82	161	139
	Baseline	5.3 (1.6)	5.3 (1.4)	5.6 (1.9)	5.6 (2.2)
	Week 12 (LOCF)	5.6 (1.9)	3.9 (0.9)	4.1 (1.1)	4.0 (1.0)
	% Change	5.7 (1.8, 9.6)	−24.1 (−28.1, −20.2) ***	−21.5 (−24.3, −18.7) ***	−22.7 (−25.8, −19.7) ***
Fibrinogen (mg/dL)	*n*	120	82	202	172
	Baseline	289.4 (45.8)	291.3 (64.5)	295.1 (52.6)	293.5 (51.9)
	Week 12 (LOCF)	292.7 (50.2)	247.6 (50.6)	251.8 (51.5)	230.1 (47.0)
	Change	1.6 (−5.8, 9.1)	−44.4 (−53.5, −35.4) ***	−42.2 (−48.0, −36.5) ***	−63.1 (−69.4, −56.9) ***
FGF21 (pg/mL)	*n*	83	76	137	125
	Baseline	423.6 (215.0)	404.5 (281.6)	548.7 (647.1)	579.0 (973.6)
	Week 12 (LOCF)	430.8 (206.3)	678.8 (531.9)	732.2 (420.6)	870.0 (669.0)
	Change	−43.0 (−134.7, 48.8)	212.7 (116.8, 308.7) ***	208.5 (137.2, 279.9) ***	334.3 (259.5, 409.0) ***

Data are presented as mean (standard deviation) for baseline and week 12 (LOCF), and least square means (95% confidence interval) for % change or change. * *p* < 0.05, ** *p* < 0.01, *** *p* < 0.001 vs. placebo by ANCOVA with baseline as covariant. FAS, full analysis set; TG, triglyceride; HDL-C, high-density lipoprotein-cholesterol; LDL-C, low-density lipoprotein-cholesterol; TC, total cholesterol; RemL-C, remnant lipoprotein-cholesterol; Apo, apolipoprotein; FGF, fibroblast growth factor; LOCF, last observation carried forward.

**Table 3:** Changes in lipid parameters from baseline to Week 12 as measured by HPLC (FAS).

**(A) With Statin**					
**Parameter**		**Placebo**		**Pemafibrate**	
			**0.1 mg/day**	**0.2 mg/day**	**0.4 mg/day**
CM-C	*n*	174	45	370	71
(mmol/L)	Baseline	0.203 (0.202)	0.220 (0.185)	0.196 (0.172)	0.205 (0.200)
	Week 12 (LOCF)	0.174 (0.254)	0.072 (0.078)	0.050 (0.061)	0.050 (0.074)
	% Change	23.8 (11.5, 36.0)	−50.4 (−74.6, −26.3) ***	−64.1 (−72.5, −55.7) ***	−62.3 (−81.5, −43.1) ***
VLDL-C	*n*	174	45	370	71
(mmol/L)	Baseline	1.315 (0.391)	1.399 (0.429)	1.314 (0.372)	1.316 (0.385)
	Week 12 (LOCF)	1.271 (0.434)	1.047 (0.373)	0.904 (0.280)	0.947 (0.397)
	% Change	−0.5 (−3.8, 2.7)	−20.5 (−26.9, −14.2) ***	−28.7 (−31.0, −26.5) ***	−26.5 (−31.6, −21.4) ***
Large LDL-C	*n*	174	45	370	71
(mmol/L)	Baseline	0.490 (0.150)	0.532 (0.122)	0.485 (0.158)	0.511 (0.157)
	Week 12 (LOCF)	0.499 (0.161)	0.687 (0.184)	0.691 (0.178)	0.762 (0.198)
	% Change	4.3 (−0.8, 9.4)	37.0 (26.9, 47.1) ***	51.1 (47.5, 54.6) ***	60.2 (52.1, 68.2) ***
Medium LDL-C	*n*	174	45	370	71
(mmol/L)	Baseline	0.975 (0.278)	1.050 (0.198)	0.952 (0.297)	1.033 (0.224)
	Week 12 (LOCF)	0.966 (0.276)	1.140 (0.266)	1.119 (0.269)	1.177 (0.308)
	% Change	1.0 (−3.4, 5.4)	15.3 (6.6, 23.9) **	24.8 (21.7, 27.8) ***	21.4 (14.5, 28.2) ***
Small LDL-C	*n*	174	45	370	71
(mmol/L)	Baseline	0.642 (0.183)	0.692 (0.134)	0.631 (0.191)	0.678 (0.153)
	Week 12 (LOCF)	0.623 (0.173)	0.619 (0.162)	0.574 (0.174)	0.563 (0.184)
	% Change	−1.2 (−5.0, 2.6)	−6.0 (−13.5, 1.5)	−5.1 (−7.7, −2.5)	−12.2 (−18.2, −6.2) **
Very small LDL-C	*n*	174	45	370	71
(mmol/L)	Baseline	0.270 (0.082)	0.306 (0.067)	0.269 (0.082)	0.287 (0.077)
	Week 12 (LOCF)	0.261 (0.077)	0.262 (0.079)	0.229 (0.070)	0.223 (0.069)
	% Change	−1.5 (−4.9, 1.9)	−9.6 (−16.3, −3.0) *	−12.3 (−14.6, −10.0) ***	−17.3 (−22.6, −12.1) ***
Very large HDL-C	*n*	174	45	370	71
(mmol/L)	Baseline	0.054 (0.017)	0.061 (0.017)	0.055 (0.019)	0.053 (0.013)
	Week 12 (LOCF)	0.054 (0.017)	0.059 (0.018)	0.053 (0.018)	0.047 (0.009)
	% Change	−0.1 (−2.8, 2.6)	−0.7 (−6.1, 4.6)	−2.2 (−4.1, −0.4)	−10.8 (−15.0, −6.6) ***
**(A) With Statin**					
**Parameter**		**Placebo**		**Pemafibrate**	
			**0.1 mg/day**	**0.2 mg/day**	**0.4 mg/day**
Large HDL-C	*n*	174	45	370	71
(mmol/L)	Baseline	0.144 (0.074)	0.159 (0.064)	0.151 (0.081)	0.139 (0.066)
	Week 12 (LOCF)	0.146 (0.074)	0.162 (0.081)	0.137 (0.085)	0.101 (0.061)
	% Change	4.0 (−0.2, 8.2)	2.1 (−6.1, 10.3)	−8.0 (−10.9, −5.2) ***	−26.3 (−32.9, −19.8) ***
Medium HDL-C	*n*	174	45	370	71
(mmol/L)	Baseline	0.361 (0.104)	0.363 (0.086)	0.363 (0.108)	0.363 (0.089)
	Week 12 (LOCF)	0.363 (0.107)	0.438 (0.128)	0.437 (0.138)	0.416 (0.118)
	% Change	1.5 (−1.7, 4.6)	20.3 (14.2, 26.5) ***	21.6 (19.5, 23.8) ***	16.2 (11.3, 21.1) ***
Small HDL-C	*n*	174	45	370	71
(mmol/L)	Baseline	0.377 (0.077)	0.379 (0.066)	0.374 (0.074)	0.377 (0.066)
	Week 12 (LOCF)	0.378 (0.075)	0.455 (0.088)	0.473 (0.083)	0.476 (0.078)
	% Change	1.2 (−1.3, 3.8)	21.9 (16.9, 26.8) ***	28.2 (26.5, 30.0) ***	28.7 (24.8, 32.7) ***
Very small HDL-C	*n*	174	45	370	71
(mmol/L)	Baseline	0.171 (0.037)	0.185 (0.036)	0.172 (0.033)	0.172 (0.037)
	Week 12 (LOCF)	0.172 (0.037)	0.210 (0.045)	0.204 (0.037)	0.203 (0.039)
	% Change	1.3 (−1.3, 4.0)	17.9 (12.6, 23.3) ***	20.3 (18.4, 22.1) ***	20.5 (16.3, 24.7) ***
**(B) Without Statin**					
**Parameter**		**Placebo**		**Pemafibrate**	
			**0.1 mg/day**	**0.2 mg/day**	**0.4 mg/day**
CM-C	*n*	110	82	199	150
(mmol/L)	Baseline	0.221 (0.175)	0.224 (0.177)	0.250 (0.227)	0.224 (0.218)
	Week 12 (LOCF)	0.212 (0.189)	0.072 (0.081)	0.065 (0.072)	0.053 (0.058)
	% Change	24.5 (13.3, 35.7)	−60.8 (−73.8, −47.8) ***	−62.4 (−70.7, −54.1) ***	−62.3 (−71.9, −52.7) ***
VLDL-C	*n*	110	82	199	150
(mmol/L)	Baseline	1.554 (0.393)	1.492 (0.382)	1.523 (0.430)	1.530 (0.466)
	Week 12 (LOCF)	1.477 (0.435)	1.121 (0.327)	1.113 (0.377)	1.104 (0.346)
	% Change	−3.3 (−7.0, 0.5)	−24.0 (−28.3, −19.6) ***	−25.5 (−28.3, −22.7) ***	−24.5 (−27.8, −21.3) ***
Large LDL-C	*n*	110	82	199	150
(mmol/L)	Baseline	0.565 (0.186)	0.542 (0.200)	0.555 (0.199)	0.559 (0.192)
	Week 12 (LOCF)	0.558 (0.178)	0.730 (0.216)	0.811 (0.210)	0.872 (0.228)
	% Change	4.6 (−3.1, 12.4)	40.6 (31.5, 49.6) ***	60.0 (54.2, 65.8) ***	68.0 (61.3, 74.6) ***
**(B) Without Statin**					
**Parameter**		**Placebo**		**Pemafibrate**	
			**0.1 mg/day**	**0.2 mg/day**	**0.4 mg/day**
Medium LDL-C	*n*	110	82	199	150
(mmol/L)	Baseline	1.234 (0.353)	1.140 (0.344)	1.187 (0.383)	1.162 (0.371)
	Week 12 (LOCF)	1.200 (0.309)	1.317 (0.327)	1.404 (0.352)	1.392 (0.327)
	% Change	4.3 (−2.5, 11.1)	19.0 (11.1, 26.9) **	30.3 (25.3, 35.4) ***	27.2 (21.4, 33.1) ***
Small LDL-C	*n*	110	82	199	150
(mmol/L)	Baseline	0.815 (0.212)	0.750 (0.217)	0.773 (0.251)	0.750 (0.229)
	Week 12 (LOCF)	0.782 (0.205)	0.696 (0.211)	0.686 (0.253)	0.641 (0.201)
	% Change	0.0 (−5.1, 5.1)	−5.0 (−10.9, 0.8)	−6.8 (−10.6, −3.0) *	−11.5 (−15.8, −7.1) ***
Very small LDL-C	*n*	110	82	199	150
(mmol/L)	Baseline	0.338 (0.099)	0.315 (0.094)	0.317 (0.110)	0.314 (0.101)
	Week 12 (LOCF)	0.321 (0.096)	0.278 (0.091)	0.271 (0.110)	0.257 (0.084)
	% Change	−2.3 (−6.5, 1.8)	−10.0 (−14.8, −5.2) *	−12.5 (−15.6, −9.4) ***	−15.8 (−19.4, −12.3) ***
Very large HDL-C	*n*	110	82	199	150
(mmol/L)	Baseline	0.052 (0.020)	0.051 (0.012)	0.049 (0.013)	0.062 (0.145)
	Week 12 (LOCF)	0.051 (0.018)	0.053 (0.014)	0.050 (0.014)	0.049 (0.040)
	% Change	−1.0 (−4.0, 2.0)	4.0 (0.5, 7.4) *	2.2 (0.0, 4.4)	−5.3 (−7.9, −2.7) *
Large HDL-C	*n*	110	82	199	150
(mmol/L)	Baseline	0.108 (0.079)	0.108 (0.042)	0.105 (0.052)	0.119 (0.158)
	Week 12 (LOCF)	0.107 (0.081)	0.117 (0.068)	0.096 (0.061)	0.088 (0.140)
	% Change	2.6 (−3.6, 8.7)	6.4 (−0.7, 13.5)	−7.3 (−11.9, −2.8) *	−24.8 (−30.0, −19.5) ***
Medium HDL-C	*n*	110	82	199	150
(mmol/L)	Baseline	0.315 (0.079)	0.309 (0.068)	0.313 (0.083)	0.308 (0.081)
	Week 12 (LOCF)	0.314 (0.079)	0.380 (0.091)	0.384 (0.105)	0.371 (0.120)
	% Change	1.9 (−2.6, 6.4)	24.2 (19.0, 29.3) ***	24.9 (21.5, 28.2) ***	21.5 (17.6, 25.3) ***
Small HDL-C	*n*	110	82	199	150
(mmol/L)	Baseline	0.360 (0.062)	0.333 (0.058)	0.348 (0.071)	0.342 (0.062)
	Week 12 (LOCF)	0.361 (0.064)	0.406 (0.065)	0.447 (0.082)	0.454 (0.076)
	% Change	3.2 (−0.3, 6.8)	21.4 (17.3, 25.5) ***	31.5 (28.9, 34.1) ***	34.5 (31.5, 37.5) ***
**(B) Without Statin**					
**Parameter**		**Placebo**		**Pemafibrate**	
			**0.1 mg/day**	**0.2 mg/day**	**0.4 mg/day**
Very small HDL-C	*n*	110	82	199	150
(mmol/L)	Baseline	0.162 (0.032)	0.155 (0.033)	0.152 (0.034)	0.154 (0.034)
	Week 12 (LOCF)	0.160 (0.034)	0.177 (0.041)	0.184 (0.039)	0.190 (0.041)
	% Change	1.2 (−2.4, 4.9)	15.0 (10.8, 19.1) ***	22.1 (19.4, 24.7) ***	25.5 (22.4, 28.6) ***

Data are presented as mean (standard deviation) for baseline and week 12 (LOCF), and least square means (95% confidence interval) for % change or change. * *p* < 0.05, ** *p* < 0.01, *** *p* < 0.001 vs. placebo by ANCOVA with baseline as covariant. HPLC, high-performance liquid chromatography; FAS, full analysis set; CM-C, chylomicron-cholesterol; VLDL-C, very-low-density lipoprotein-cholesterol; LDL-C, low-density lipoprotein-cholesterol; HDL-C, high-density lipoprotein-cholesterol; LOCF, last observation carried forward.

**Table 4:** Summary of AEs and ADRs (SAS).

**(A) With Statin**				
**Parameter**	**Placebo**		**Pemafibrate**	
		**0.1 mg/day**	**0.2 mg/day**	**0.4 mg/day**
*n*	178	45	382	72
AE				
Total	73 (41.0)	29 (64.4)	164 (42.9)	34 (47.2)
Serious	2 (1.1)	2 (4.4)	6 (1.6)	0
Leading to withdrawal	2 (1.1)	2 (4.4)	12 (3.1)	0
ADR				
Total	17 (9.6)	3 (6.7)	36 (9.4)	2 (2.8)
Serious	1 (0.6)	0	2 (0.5)	0
Leading to withdrawal	2 (1.1)	1 (2.2)	11 (2.9)	0
*n*	178	45	382	72
AST ≥ ULN × 3	0 ^1^	0	1 (0.3)	1 (1.4)
ALT ≥ ULN × 3	1 (0.6)	1 (2.2)	0	1 (1.4)
sCr ≥ ULN	37 (20.8)	8 (17.8)	61 (16.0)	11 (15.3)
CK ≥ 2.5 and < ULN × 5	4 (2.2)	2 (4.4)	7 (1.8)	2 (2.8)
CK ≥ 5 and < ULN × 10	0	0	3 (0.8)	0
CK ≥ ULN × 10	0	0	1 (0.3)	0
**(B) Without Statin**				
**Parameter**	**Placebo**		**Pemafibrate**	
		**0.1 mg/day**	**0.2 mg/day**	**0.4 mg/day**
*n*	120	82	202	174
AE				
Total	55 (45.8)	27 (32.9)	78 (38.6)	60 (34.5)
Serious	0	1 (1.2)	4 (2.0)	2 (1.1)
Leading to withdrawal	0	2 (2.4)	3 (1.5)	6 (3.4)
ADR				
Total	10 (8.3)	3 (3.7)	14 (6.9)	16 (9.2)
Serious	0	0	1 (0.5)	1 (0.6)
Leading to withdrawal	0	1 (1.2)	2 (1.0)	3 (1.7)
*n*	120	82	202	173
AST ≥ ULN × 3	0	0	0	1 (0.6)
ALT ≥ ULN × 3	0	0	0	0
sCr ≥ ULN	16 (13.3)	15 (18.3)	34 (16.8)	22 (12.7)
CK ≥ 2.5 and < ULN × 5	1 (0.8)	2 (2.4)	3 (1.5)	0
CK ≥ 5 and < ULN × 10	1 (0.8)	0	0	1 (0.6)
CK ≥ ULN × 10	0	0	0	1 (0.6)

Data are presented as the number of patients (percentage). ^1^ *n* = 177. AE, adverse event; ADR, adverse drug reaction; SAS, safety analysis set; AST, aspartate aminotransferase; ULN, upper limit of normal; ALT, alanine aminotransferase; sCr, serum creatinine; CK, creatine kinase.

**Table 5:** Adverse events with concomitant statin treatment stratified by the presence or absence of renal dysfunction (SAS).

**(A) Baseline eGFR ^1^ ≥ 60 mL/min/1.73 m^2^**				
**Parameter**	**Placebo**		**Pemafibrate**	
		**0.1 mg/day**	**0.2 mg/day**	**0.4 mg/day**
*n*	143	39	335	64
AE				
Total	55 (38.5)	26 (66.7)	141 (42.1)	31 (48.4)
Serious	1 (0.7)	2 (5.1)	3 (0.9)	0
Leading to withdrawal	0	2 (5.1)	10 (3.0)	0
ADR				
Total	9 (6.3)	3 (7.7)	28 (8.4)	2 (3.1)
Serious	0	0	1 (0.3)	0
Leading to withdrawal	0	1 (2.6)	9 (2.7)	0
*n*	143	39	335	64
AST ≥ ULN × 3	0 ^2^	0	0	1 (1.6)
ALT ≥ ULN × 3	1 (0.7)	1 (2.6)	0	1 (1.6)
sCr ≥ ULN	5 (3.5)	2 (5.1)	21 (6.3)	4 (6.3)
CK ≥ 2.5 and < ULN × 5	1 (0.7)	2 (5.1)	6 (1.8)	2 (3.1)
CK ≥ 5 and < ULN × 10	0	0	2 (0.6)	0
CK ≥ ULN × 10	0	0	1 (0.3)	0
**(B) Baseline eGFR ^1^ < 60 mL/min/1.73 m^2^**				
**Parameter**	**Placebo**		**Pemafibrate**	
		**0.1 mg/day**	**0.2 mg/day**	**0.4 mg/day**
*n*	35	6	47	8
AE				
Total	18 (51.4)	3 (50.0)	23 (48.9)	3 (37.5)
Serious	1 (2.9)	0	3 (6.4)	0
Leading to withdrawal	2 (5.7)	0	2 (4.3)	0
ADR				
Total	8 (22.9)	0	8 (17.0)	0
Serious	1 (2.9)	0	1 (2.1)	0
Leading to withdrawal	2 (5.7)	0	2 (4.3)	0
*n*	35	6	47	8
AST ≥ ULN × 3	0	0	1 (2.1)	0
ALT ≥ ULN × 3	0	0	0	0
sCr ≥ ULN	32 (91.4)	6 (100.0)	40 (85.1)	7 (87.5)
CK ≥ 2.5 and < ULN × 5	3 (8.6)	0	1 (2.1)	0
CK ≥ 5 and < ULN × 10	0	0	1 (2.1)	0
CK ≥ ULN × 10	0	0	0	0

Data are presented as the number of patients (percentage). ^1^ eGFR_male_ = 194 × sCr^−1.094^ × age^−0.287^, eGFR_female_ = 194 × sCr^−1.094^ × age^-0.287^ × 0.739. ^2^ *n* = 142. AE, adverse event; ADR, adverse drug reaction; SAS, safety analysis set; eGFR, estimated glomerular filtration rate; AST, aspartate aminotransferase; ULN, upper limit of normal; ALT, alanine aminotransferase; sCr, serum creatinine; CK, creatine kinase.

**Table 6:** Changes in safety parameters from baseline to Week 12 (SAS).

**(A) With Statin**					
**Parameter**		**Placebo**		**Pemafibrate**	
			**0.1 mg/day**	**0.2 mg/day**	**0.4 mg/day**
sCr (mg/dL)	*n*	174	44	368	71
	Baseline	0.81 (0.18)	0.80 (0.18)	0.79 (0.16)	0.80 (0.17)
	Week 12	0.80 (0.18)	0.81 (0.18)	0.82 (0.18)	0.85 (0.19)
	Change	0.00 (−0.01, 0.01)	0.01 (−0.01, 0.03)	0.02 (0.02, 0.03) ***	0.05 (0.04, 0.07) ***
eGFR ^1^	*n*	174	44	368	71
(mL/min/1.73 m^2^)	Baseline	76.8 (18.5)	78.1 (17.8)	77.9 (16.5)	78.2 (17.1)
	Week 12	76.9 (18.1)	77.1 (16.7)	75.7 (16.1)	73.3 (17.2)
	Change	0.0 (−1.0, 1.0)	−0.9 (−2.9, 1.1)	−2.2 (−2.8, −1.5) ***	−4.8 (−6.3, −3.2) ***
CK (U/L)	*n*	174	44	368	71
	Baseline	145.2 (128.9)	151.5 (108.1)	132.8 (105.4)	137.9 (71.0)
	Week 12	129.0 (59.5)	154.0 (118.5)	140.7 (125.8)	151.9 (111.7)
	Change	−11.2 (−26.8, 4.4)	11.9 (−19.1, 42.9)	4.4 (−6.3, 15.1)	14.0 (−10.4, 38.4)
AST (U/L)	*n*	169	44	362	70
	Baseline	31.2 (10.0)	30.5 (8.1)	31.5 (13.7)	31.9 (17.8)
	Week 12	31.6 (11.5)	32.4 (15.4)	30.6 (13.0)	29.6 (7.5)
	Change	0.3 (−1.2, 1.8)	1.5 (−1.4, 4.3)	−0.8 (−1.8, 0.2)	−2.1 (−4.4, 0.2)
ALT (U/L)	*n*	174	44	368	71
	Baseline	38.3 (18.0)	37.5 (17.0)	38.5 (21.1)	38.1 (20.6)
	Week 12	38.9 (20.8)	36.5 (27.0)	29.5 (18.2)	27.4 (14.1)
	Change	0.6 (−1.5, 2.7)	−1.3 (−5.5, 2.9)	−8.9 (−10.4, −7.5) ***	−10.8 (−14.2, −7.5) ***
γ-GT (U/L)	*n*	174	44	368	71
	Baseline	84.7 (90.7)	80.9 (70.1)	82.1 (74.5)	75.8 (99.9)
	Week 12	89.0 (105.6)	51.3 (38.2)	45.9 (46.9)	31.2 (25.0)
	Change	5.3 (−0.8, 11.3)	−30.0 (−42.0, −18.0) ***	−36.2 (−40.3, −32.0) ***	−46.9 (−56.3, −37.4) ***
ALP (U/L)	*n*	174	44	368	71
	Baseline	231.9 (61.4)	231.4 (59.0)	242.4 (72.5)	221.1 (53.8)
	Week 12	229.8 (62.4)	177.8 (49.5)	165.1 (54.4)	131.6 (32.1)
	Change	−3.5 (−8.0, 1.0)	−55.2 (−64.2, −46.2) ***	−75.4 (−78.5, −72.3) ***	−94.5 (−101.6, −87.4) ***
Total bilirubin	*n*	174	44	368	71
(mg/dL)	Baseline	0.77 (0.36)	0.75 (0.30)	0.76 (0.30)	0.75 (0.40)
	Week 12	0.79 (0.32)	0.66 (0.27)	0.64 (0.21)	0.59 (0.19)
	Change	0.02 (−0.01, 0.05)	−0.10 (−0.15, −0.04) ***	−0.12 (−0.14, −0.10) ***	−0.16 (−0.21, −0.12) ***
**(B) Without Statin**					
**Parameter**		**Placebo**		**Pemafibrate**	
			**0.1 mg/day**	**0.2 mg/day**	**0.4 mg/day**
sCr (mg/dL)	*n*	118	79	196	165
	Baseline	0.82 (0.17)	0.87 (0.14)	0.82 (0.16)	0.83 (0.16)
	Week 12	0.81 (0.17)	0.87 (0.14)	0.84 (0.23)	0.86 (0.25)
	Change	−0.01 (−0.03, 0.01)	−0.01 (−0.03, 0.02)	0.02 (0.01, 0.04) *	0.04 (0.02, 0.05) ***
eGFR ^1^	*n*	118	79	196	165
(mL/min/1.73 m^2^)	Baseline	78.6 (15.5)	75.9 (14.5)	78.9 (15.5)	78.9 (15.5)
	Week 12	79.9 (15.8)	75.7 (14.4)	78.1 (17.3)	76.9 (17.5)
	Change	1.3 (0.1, 2.6)	−0.2 (−1.8, 1.3)	−0.7 (−1.7, 0.2) *	−2.0 (−3.1, −0.9) ***
CK (U/L)	*n*	118	79	196	165
	Baseline	125.4 (69.0)	148.3 (209.9)	120.6 (118.2)	125.8 (89.5)
	Week 12	127.4 (84.3)	128.4 (60.7)	119.3 (55.4)	124.5 (60.7)
	Change	0.6 (−10.6, 11.8)	−2.0 (−15.7, 11.8)	−6.8 (−15.5, 1.9)	−2.4 (−11.8, 7.1)
AST (U/L)	*n*	117	78	193	162
	Baseline	28.1 (11.8)	27.1 (8.4)	27.8 (11.3)	27.6 (9.4)
	Week 12	29.0 (13.3)	27.4 (9.9)	28.0 (10.1)	29.6 (10.5)
	Change	1.0 (−0.4, 2.5)	0.1 (−1.6, 1.9)	0.2 (−0.9, 1.3)	2.0 (0.8, 3.3)
ALT (U/L)	*n*	118	79	196	165
	Baseline	36.4 (19.1)	34.7 (16.8)	33.9 (18.8)	33.6 (17.7)
	Week 12	37.8 (21.9)	30.4 (17.2)	28.7 (15.5)	28.1 (15.4)
	Change	2.0 (−0.2, 4.1)	−4.2 (−6.8, −1.6) ***	−5.4 (−7.0, −3.7) ***	−5.8 (−7.6, −4.0) ***
γ-GT (U/L)	*n*	118	79	196	165
	Baseline	65.9 (61.6)	56.6 (39.1)	65.7 (54.8)	57.6 (47.8)
	Week 12	65.3 (61.9)	38.3 (23.9)	38.3 (41.8)	28.4 (18.5)
	Change	1.0 (−3.6, 5.6)	−20.5 (−26.2, −14.9) ***	−25.9 (−29.4, −22.3) ***	−31.0 (−35.0, −27.1) ***
**(B) Without Statin**					
**Parameter**		**Placebo**		**Pemafibrate**	
			**0.1 mg/day**	**0.2 mg/day**	**0.4 mg/day**
ALP (U/L)	*n*	118	79	196	165
	Baseline	236.9 (62.2)	226.9 (55.6)	229.5 (62.0)	225.6 (55.9)
	Week 12	237.7 (61.4)	176.5 (47.9)	164.1 (49.2)	145.0 (42.2)
	Change	3.2 (−2.2, 8.7)	−51.3 (−57.9, −44.6) ***	−65.5 (−69.7, −61.2) ***	−81.9 (−86.5, −77.3) ***
Total bilirubin	*n*	118	79	196	165
(mg/dL)	Baseline	0.85 (0.31)	0.78 (0.31)	0.81 (0.34)	0.75 (0.28)
	Week 12	0.82 (0.32)	0.68 (0.22)	0.67 (0.26)	0.64 (0.21)
	Change	0.00 (−0.04, 0.03)	−0.11 (−0.15, −0.07) ***	−0.13 (−0.16, −0.10) ***	−0.13 (−0.16, −0.10) ***

Data are presented as mean (standard deviation) for baseline and week 12, and least square means (95% confidence interval) for % change or change. * *p* < 0.05, ** *p* < 0.01, *** *p* < 0.001 vs. placebo by ANCOVA with baseline as covariant. ^1^ eGFR_male_ = 194 × sCr^−1.094^ × age^−0.287^, eGFR_female_ = 194 × sCr^−1.094^ × age^−0.287^ × 0.739. SAS, safety analysis set; sCr, serum creatinine; eGFR, estimated glomerular filtration rate; CK, creatine kinase; AST, aspartate aminotransferase; ALT, alanine aminotransferase; γ-GT, gamma-glutamyltransferase; ALP, alkaline phosphatase.

## Supplementary Materials

Supplementary materials can be found at https://www.mdpi.com/1422-0067/20/22/5537/s1.

## Abbreviations

Abbreviation	Expansion
ABCA1	ATP-binding cassette transporter A1
ABCG1	ATP-binding cassette transporter G1
ADRs	adverse drug reactions
AEs	adverse events
ALP	alkaline phosphatase
ALT	alanine aminotransferase
ANCOVA	analysis of covariance
ATP	adenosine triphosphate
Apo	apolipoprotein
AST	aspartate aminotransferase
BMI	body mass index
CEC	cholesterol efflux capacity
CK	creatine kinase
CKD	chronic kidney disease
CM-C	chylomicron-cholesterol
CV	cardiovascular
eGFR	estimated glomerular filtration rate
FAS	full analysis set
FDA	Food and Drug Administration
FGF21	fibroblast growth factor 21
Hb	hemoglobin
HDL-C	high-density lipoprotein-cholesterol
HPLC	high-performance liquid chromatography
IL	interleukin
LDL-C	low-density lipoprotein-cholesterol
LOCF	last observation carried forward
LPL	lipoprotein lipase
LS	least squares
mRNA	messenger ribonucleic acid
NPC1L1	Niemann-Pick C 1-Like-1
PPARα	peroxisome proliferator-activated receptor α
RemL-C	remnant lipoprotein-cholesterol
sCr	serum creatinine
SPPARMα	selective peroxisome proliferator-activated receptor α modulator
TC	total cholesterol
TG	triglyceride
VCAM-1	vascular cell adhesion molecule-1
VLDL-C	very-low-density lipoprotein-cholesterol
γ-GT	gamma-glutamyltransferase
