# Supplementary material for: Efficacy and Safety of Pemafibrate, a Novel Selective Peroxisome Proliferator-Activated Receptor α Modulator (SPPARMα): Pooled Analysis of Phase 2 and 3 Studies in Dyslipidemic Patients with or without Statin Combination
Source: Int J Mol Sci. 2019 Nov 6;20(22):5537. doi: 10.3390/ijms20225537 (PMC6888510; doi:10.3390/ijms20225537)
Supplement: Supplementary file 1 [file ijms-20-05537-s001.pdf]

Article

Efficacy and safety of pemafibrate, a novel selective peroxisome proliferator-activated receptor α modulator (SPPARMα): pooled analysis of phase 2 and 3 studies in dyslipidemic patients with or without statin combination

Supplementary Materials

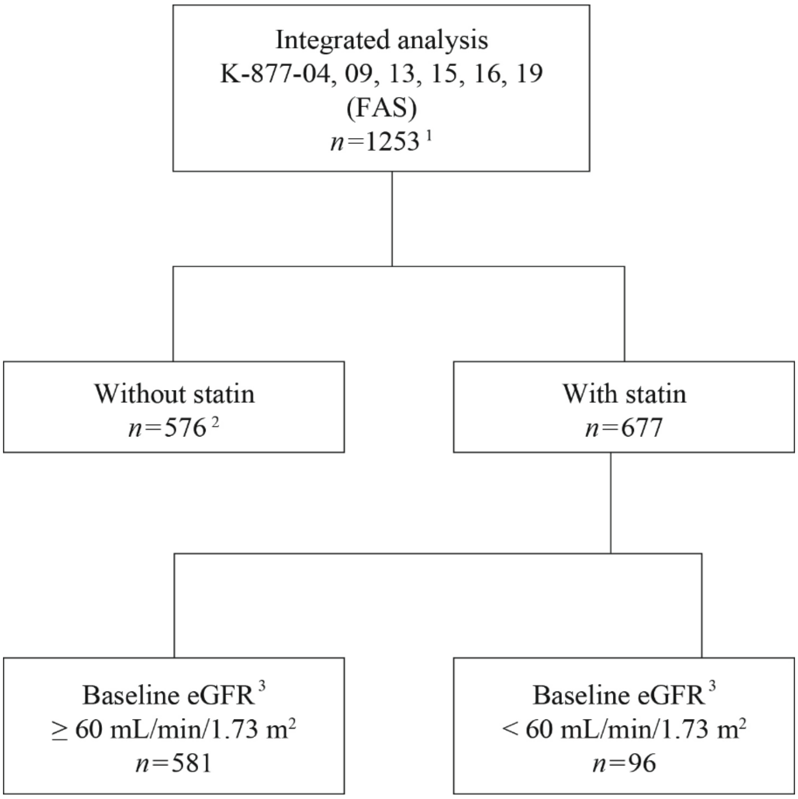

Figure S1. Patient population.

[1] Safety analysis set: n = 1255, [2] Safety analysis set: n = 578, [3] $eGFR_{male} = 194 \times sCr^{-1.094} \times age^{-0.287}$, $eGFR_{female} = 194 \times sCr^{-1.094} \times age^{-0.287} \times 0.739$. FAS, full analysis set; eGFR, estimated glomerular filtration rate; sCr, serum creatinine.

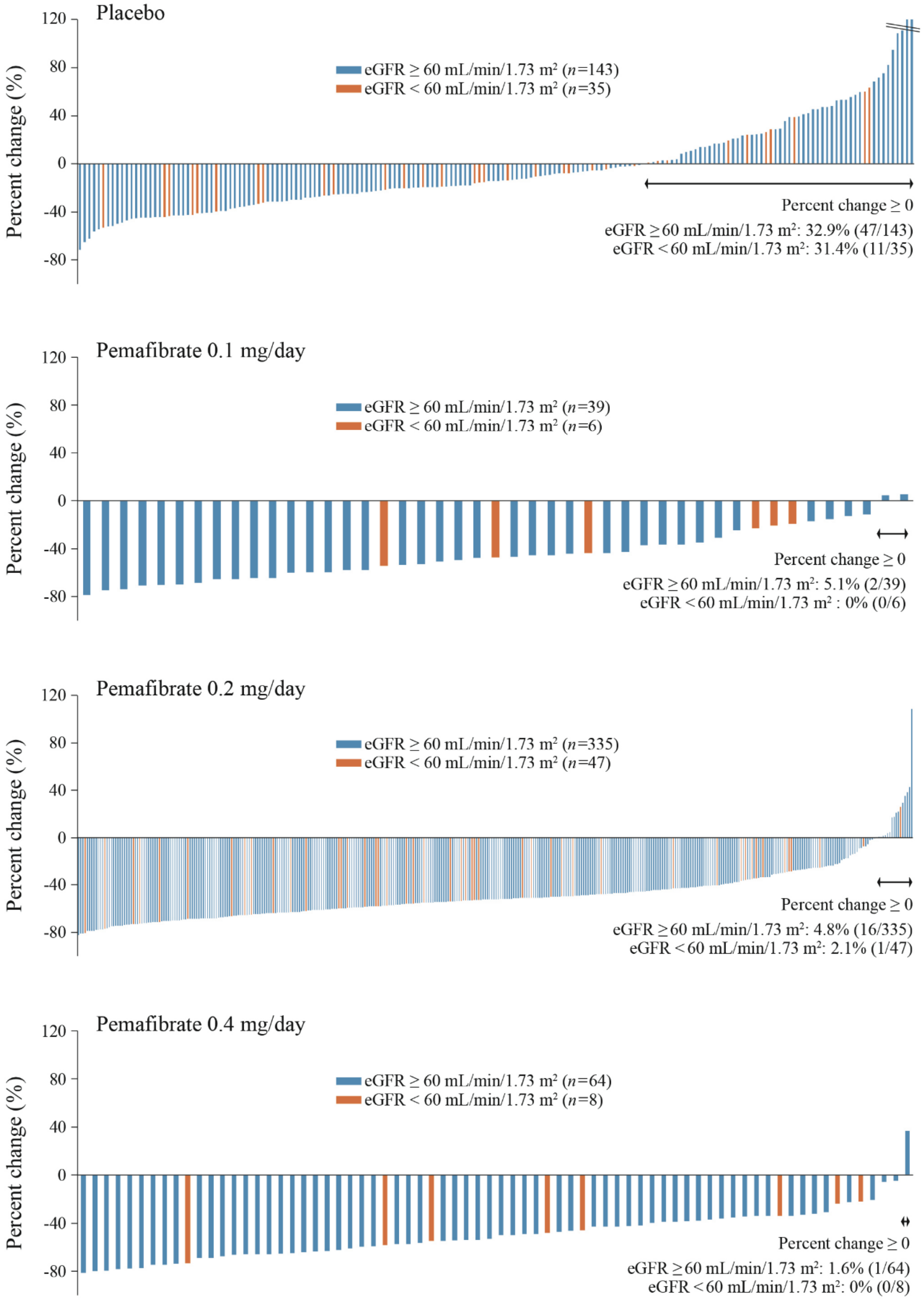

Figure S2. Change in TG in patients treated concomitantly with statin from baseline to week 12, stratified by presence or absence of renal dysfunction.

$eGFR_{male} = 194 \times sCr^{-1.094} \times age^{-0.287}$, $eGFR_{female} = 194 \times sCr^{-1.094} \times age^{-0.287} \times 0.739$. TG, triglyceride; eGFR, estimated glomerular filtration rate; sCr, serum creatinine.

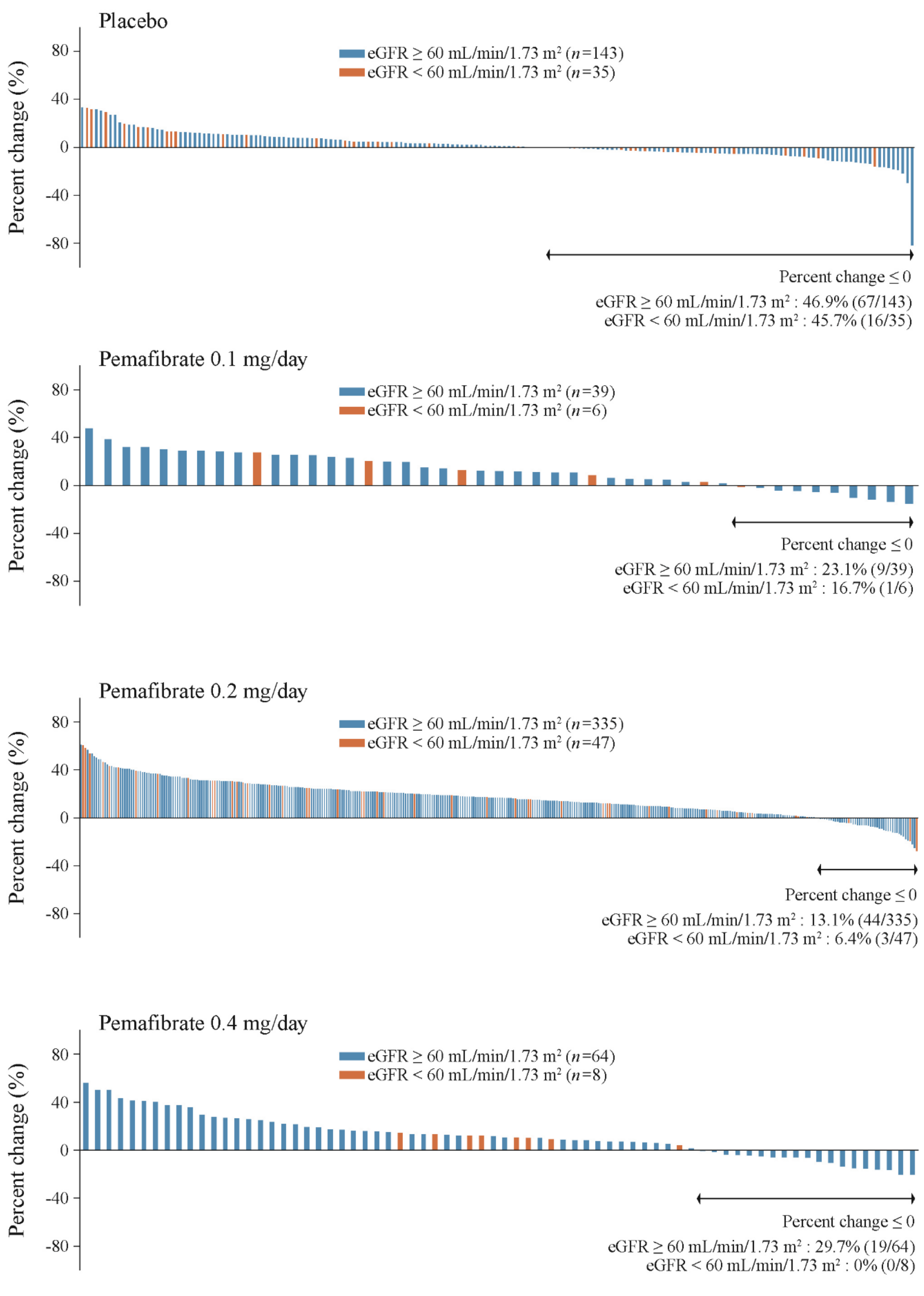

Figure S3. Change in HDL-C in patients treated concomitantly with statin from baseline to week 12, stratified by presence or absence of renal dysfunction.

$eGFR_{male} = 194 \times sCr^{-1.094} \times age^{-0.287}$, $eGFR_{female} = 194 \times sCr^{-1.094} \times age^{-0.287} \times 0.739$. HDL-C, high-density lipoprotein-cholesterol; eGFR, estimated glomerular filtration rate; sCr, serum creatinine.

Table S1. Characteristics of patients at baseline stratified by renal dysfunction (with statin) (FAS).

Parameter	Baseline eGFR[1] ≥ 60 mL/min/1.73 m²					Baseline eGFR[1] < 60 mL/min/1.73 m²				
	Placebo	Pemafibrate			All	Placebo	Pemafibrate			All
		0.1 mg/day	0.2 mg/day	0.4 mg/day			0.1 mg/day	0.2 mg/day	0.4 mg/day	
n	143	39	335	64	581	35	6	47	8	96
Age (years)	54.8 (10.9)	54.3 (10.3)	55.9 (11.1)	54.3 (10.7)	55.4 (10.9)	65.4 (9.1)	59.8 (9.5)	64.0 (8.9)	66.5 (8.4)	64.4 (9.0)
Age ≥65 years	27 (18.9)	5 (12.8)	77 (23.0)	9 (14.1)	118 (20.3)	17 (48.6)	2 (33.3)	26 (55.3)	4 (50.0)	49 (51.0)
Sex, Female	27 (18.9)	8 (20.5)	62 (18.5)	10 (15.6)	107 (18.4)	11 (31.4)	1 (16.7)	11 (23.4)	4 (50.0)	27 (28.1)
Body weight (kg)	75.46 (14.31)	77.10 (12.67)	74.75 (14.19) [2]	74.10 (13.35)	75.01 (14.02) [3]	71.78 (12.69)	67.06 (8.34)	71.48 (13.54)	64.46 (5.48)	70.73 (12.53)
BMI (kg/m²)	27.31 (3.79)	27.77 (3.71)	27.25 (4.11) [2]	26.45 (3.51)	27.21 (3.94) [3]	26.87 (3.40)	24.84 (2.58)	26.68 (3.21)	25.50 (2.74)	26.54 (3.22)
BMI ≥25 kg/m²	98 (68.5)	31 (79.5)	224 (66.9)	38 (59.4)	391 (67.3)	27 (77.1)	3 (50.0)	33 (70.2)	5 (62.5)	68 (70.8)
Type 2 diabetes	52 (36.4)	13 (33.3)	135 (40.3)	31 (48.4)	231 (39.8)	20 (57.1)	2 (33.3)	19 (40.4)	8 (100.0)	49 (51.0)
Hypertension	76 (53.1)	18 (46.2)	215 (64.2)	36 (56.3)	345 (59.4)	31 (88.6)	2 (33.3)	36 (76.6)	7 (87.5)	76 (79.2)
Fatty liver	88 (61.5)	12 (30.8)	205 (61.2)	23 (35.9)	328 (56.5)	18 (51.4)	1 (16.7)	30 (63.8)	4 (50.0)	53 (55.2)
Pravastatin	18 (12.6)	0	45 (13.4)	0	63 (10.8)	4 (11.4)	0	4 (8.5)	0	8 (8.3)
Simvastatin	4 (2.8)	0	7 (2.1)	1 (1.6)	12 (2.1)	1 (2.9)	0	0	0	1 (1.0)
Fluvastatin	3 (2.1)	0	7 (2.1)	3 (4.7)	13 (2.2)	1 (2.9)	0	1 (2.1)	1 (12.5)	3 (3.1)
Atorvastatin	26 (18.2)	0	66 (19.7)	2 (3.1)	94 (16.2)	4 (11.4)	0	10 (21.3) [4]	1 (12.5)	15 (15.6)
Pitavastatin	57 (39.9)	39 (100)	119 (35.5)	53 (82.8)	268 (46.1)	16 (45.7)	6 (100)	15 (31.9)	4 (50.0)	41 (42.7)
Rosuvastatin	35 (24.5) [5]	0	91 (27.2)	5 (7.8)	131 (22.5)	9 (25.7)	0	17 (36.2) [4]	2 (25.0)	28 (29.2)
TG (mmol/L)	3.90 (1.83)	3.77 (1.38)	3.70 (1.56)	3.71 (1.36)	3.76 (1.60)	3.29 (1.06)	4.56 (1.69)	3.56 (1.22)	2.88 (1.02)	3.47 (1.21)
HDL-C (mmol/L)	1.19 (0.29)	1.26 (0.23)	1.19 (0.27)	1.17 (0.21)	1.19 (0.26)	1.15 (0.19)	1.13 (0.16)	1.16 (0.20)	1.28 (0.28)	1.16 (0.20)
LDL-C (mmol/L)	2.87 (0.79)	3.22 (0.49)	2.85 (0.79)	3.10 (0.62)	2.91 (0.77)	2.97 (0.60)	3.43 (0.68)	2.85 (0.68)	3.09 (0.57)	2.95 (0.65)
HbA1c (%)	6.34 (0.78)	6.31 (0.61)	6.44 (0.88)	6.54 (0.66)	6.42 (0.82)	6.63 (0.64)	6.35 (0.69)	6.33 (0.66)	7.04 (0.36)	6.50 (0.66)
eGFR[1] (mL/min/1.73m²)	82.2 (15.6)	82.1 (15.6)	81.3 (14.6)	81.5 (14.5)	81.6 (14.9)	52.6 (6.9)	53.1 (4.2)	52.8 (5.7)	50.3 (6.4)	52.6 (6.1)

Data are presented as mean (standard deviation) for continuous parameters and the number of patients (percentage) for categorical parameters. [1] $eGFR_{male} = 194 \times sCr^{-1.094} \times age^{-0.287}$, $eGFR_{female} = 194 \times sCr^{-1.094} \times age^{-0.287} \times 0.739$, [2] n = 334, [3] n = 580, [4] one patient switched from atorvastatin to rosuvastatin at week 2, [5] including one patient who started rosuvastatin at week 8. FAS, full analysis set; eGFR, estimated glomerular filtration rate; BMI, body mass index; TG, triglyceride; HDL-C, high-density lipoprotein-cholesterol; LDL-C, low-density lipoprotein-cholesterol; HbA1c, hemoglobin A1c; sCr, serum creatinine.

Table S2. Changes in lipoproteins, fibrinogen, and FGF21 from baseline to week 12 (with statin) (FAS).

Parameter		Baseline eGFR [1] ≥ 60 mL/min/1.73 m²		Baseline eGFR [1] < 60 mL/min/1.73 m²	
		Placebo	Pemafibrate 0.1–0.4 mg/day	Placebo	Pemafibrate 0.1–0.4 mg/day
TG (mmol/L)	*n*	143	438	35	61
	Baseline	3.90 (1.83)	3.71 (1.51)	3.29 (1.06)	3.57 (1.29)
	Week 12 (LOCF)	3.91 (5.22)	1.85 (1.02)	3.03 (1.43)	1.76 (0.71)
	% Change	−0.3 (−6.2, 5.6)	−48.4 (−51.8, −45.1) ***	−8.9 (−16.5, −1.4)	−48.0 (−53.7, −42.3) ***
HDL-C (mmol/L)	*n*	143	438	35	61
	Baseline	1.19 (0.29)	1.19 (0.26)	1.15 (0.19)	1.17 (0.21)
	Week 12 (LOCF)	1.20 (0.31)	1.37 (0.32)	1.20 (0.19)	1.38 (0.30)
	% Change	1.0 (−1.4, 3.4)	15.2 (13.8, 16.5) ***	4.8 (−0.1, 9.6)	18.3 (14.6, 22.0) ***
LDL-C (mmol/L)	*n*	143	438	35	61
	Baseline	2.87 (0.79)	2.92 (0.76)	2.97 (0.60)	2.94 (0.69)
	Week 12 (LOCF)	2.77 (0.81)	3.03 (0.74)	3.03 (0.67)	3.06 (0.89)
	% Change	−3.2 (−7.1, 0.7)	8.5 (6.2, 10.7) ***	3.5 (−4.2, 11.2)	6.6 (0.8, 12.5)
Non-HDL-C (mmol/L)	*n*	143	438	35	61
	Baseline	4.05 (0.73)	4.06 (0.73)	4.03 (0.60)	4.11 (0.70)
	Week 12 (LOCF)	3.93 (1.46)	3.68 (0.81)	3.98 (0.68)	3.70 (0.96)
	% Change	−2.9 (−6.1, 0.3)	−8.6 (−10.4, −6.7) **	−0.8 (−6.2, 4.7)	−9.7 (−13.8, −5.6) *
TC (mmol/L)	*n*	143	438	35	61
	Baseline	5.24 (0.80)	5.26 (0.78)	5.18 (0.65)	5.28 (0.73)
	Week 12 (LOCF)	5.12 (1.42)	5.05 (0.78)	5.18 (0.70)	5.07 (0.96)
	% Change	−2.1 (−4.4, 0.3)	−3.2 (−4.6, −1.8)	0.3 (−4.0, 4.6)	−3.5 (−6.7, −0.2)
RemL-C (mmol/L)	*n*	36	130	8	10
	Baseline	0.69 (0.40)	0.63 (0.39)	0.50 (0.24)	0.92 (0.68)
	Week 12 (LOCF)	0.63 (0.31)	0.28 (0.21)	0.56 (0.34)	0.39 (0.29)
	% Change	12.9 (−0.7, 26.5)	−47.7 (−54.9, −40.6) ***	21.5 (−30.5, 73.4)	−45.2 (−91.3, 0.8)
ApoAI (mg/dL)	*n*	123	400	25	52
	Baseline	139.7 (22.6)	137.4 (21.0)	132.1 (16.9)	134.4 (15.0)
	Week 12 (LOCF)	137.3 (23.3)	142.3 (19.6)	135.2 (14.9)	143.9 (17.9)
	% Change	-1.3 (-2.8, 0.2)	4.0 (3.2, 4.8) ***	2.5 (-1.2, 6.3)	7.7 (5.1, 10.3) *
ApoAII (mg/dL)	*n*	123	400	25	52
	Baseline	32.4 (4.9)	32.3 (4.9)	29.9 (3.3)	30.4 (4.6)
	Week 12 (LOCF)	32.1 (5.4)	40.3 (7.3)	30.7 (3.2)	40.0 (7.5)
	% Change	−0.9 (−3.5, 1.8)	25.5 (24.0, 27.0) ***	2.7 (−4.2, 9.6)	32.5 (27.7, 37.2) ***
ApoB (mg/dL)	*n*	123	400	25	52
	Baseline	98.7 (18.4)	97.8 (18.0)	98.8 (15.5)	98.0 (19.8)

	Week 12 (LOCF)	93.2 (16.9)	91.1 (18.7)	98.0 (14.7)	90.1 (21.7)
	% Change	−4.5 (−7.3, −1.8)	−5.6 (−7.2, −4.1)	0.3 (−5.7, 6.2)	−7.4 (−11.5, −3.2) *
ApoB48 (μg/mL)	*n*	38	131	8	10
	Baseline	14.1 (10.5)	11.1 (6.7)	13.2 (9.1)	21.8 (28.1)
	Week 12 (LOCF)	14.4 (16.5)	4.6 (3.5)	13.2 (8.3)	8.2 (7.0)
	% Change	23.3 (8.6, 37.9)	−54.2 (−62.1, −46.4) ***	48.2 (−49.1, 145.5)	−46.6 (−133.4, 40.2)
ApoB100 (mg/dL)	*n*	36	130	8	10
	Baseline	103.3 (18.3)	103.9 (14.6)	100.3 (14.7)	103.3 (21.4)
	Week 12 (LOCF)	97.9 (17.1)	94.7 (18.0)	102.2 (16.6)	101.2 (30.3)
	% Change	−4.8 (−9.9, 0.2)	−7.9 (−10.6, −5.3)	2.6 (−6.9, 12.1)	−3.1 (−11.6, 5.4)
ApoCII (mg/dL)	*n*	123	400	25	52
	Baseline	8.1 (2.6)	8.2 (2.5)	8.1 (2.4)	8.4 (2.1)
	Week 12 (LOCF)	7.8 (2.1)	6.4 (2.3)	8.4 (2.8)	6.4 (1.6)
	% Change	−1.2 (−5.1, 2.6)	−20.4 (−22.6, −18.3) ***	4.7 (−3.3, 12.6)	−21.6 (−27.1, −16.1) ***
ApoCIII (mg/dL)	*n*	123	400	25	52
	Baseline	17.7 (6.4)	17.3 (5.7)	17.4 (6.6)	17.3 (5.5)
	Week 12 (LOCF)	16.8 (5.7)	11.2 (4.1)	17.6 (6.2)	11.3 (4.2)
	% Change	−1.1 (−4.8, 2.6)	−32.9 (−35.0, −30.9) ***	3.7 (−4.3, 11.6)	−32.8 (−38.3, −27.3) ***
ApoCIII/ApoCII	*n*	123	400	25	52
	Baseline	2.2 (0.4)	2.1 (0.5)	2.2 (0.6)	2.1 (0.4)
	Week 12 (LOCF)	2.2 (0.5)	1.8 (0.5)	2.2 (0.5)	1.8 (0.4)
	% Change	0.4 (−2.5, 3.2)	−14.4 (−16.0, −12.8) ***	2.0 (−5.0, 8.9)	−13.9 (−18.7, −9.1) ***
ApoE (mg/dL)	*n*	123	400	25	52
	Baseline	5.5 (2.1)	5.3 (1.8)	4.9 (1.3)	5.1 (1.4)
	Week 12 (LOCF)	5.2 (1.7)	4.0 (1.0)	5.2 (1.4)	4.0 (0.7)
	% Change	0.1 (−3.3, 3.6)	−19.2 (−21.2, −17.3) ***	5.8 (−0.3, 11.9)	−18.5 (−22.7, −14.3) ***
Fibrinogen (mg/dL)	*n*	142	437	35	61
	Baseline	282.4 (46.9)	283.6 (50.4)	299.8 (55.4)	308.3 (62.5)
	Week 12 (LOCF)	284.4 (48.8)	239.3 (48.8)	307.0 (52.2)	253.7 (76.8)
	Change	1.6 (−4.9, 8.2)	−44.2 (−47.9, −40.4) ***	4.1 (−17.5, 25.7)	−52.9 (−69.2, −36.5) ***
FGF21 (pg/mL)	*n*	38	131	8	10
	Baseline	617.0 (956.2)	502.4 (902.2)	529.3 (301.6)	622.7 (173.3)
	Week 12 (LOCF)	465.0 (230.2)	831.0 (1290.8)	541.7 (396.0)	1419.6 (987.3)
	Change	−155.7 (−359.3, 47.8)	329.7 (220.1, 439.2) ***	−28.6 (−645.9, 588.6)	829.7 (278.9, 1380.5) *

Data are presented as mean (standard deviation) for baseline and week 12 (LOCF), and least square means (95% confidence interval) for % change or change. * $p < 0.05$, ** $p < 0.01$, *** $p < 0.001$ vs. placebo by ANCOVA with baseline as covariant. [1] $eGFR_{male} = 194 \times sCr^{-1.094} \times age^{-0.287}$, $eGFR_{female} = 194 \times sCr^{-1.094} \times age^{-0.287} \times 0.739$. FAS, full analysis set; eGFR, estimated glomerular filtration rate; TG, triglyceride; HDL-C, high-density lipoprotein-cholesterol; LDL-C, low-density lipoprotein-

cholesterol; TC, total cholesterol; RemL-C, remnant lipoprotein-cholesterol; Apo, apolipoprotein; FGF, fibroblast growth factor; LOCF, last observation carried forward; sCr, serum creatinine.

Table S3. Changes in lipoproteins from baseline to week 12 (with statin) (FAS).

**Parameter**		Baseline eGFR [1] ≥ 60 mL/min/1.73 m²		Baseline eGFR [1] < 60 mL/min/1.73 m²	
		Placebo	Pemafibrate 0.1–0.4 mg/day	Placebo	Pemafibrate 0.1–0.4 mg/day
CM-C	*n*	141	428	33	58
(mmol/L)	Baseline	0.216 (0.217)	0.198 (0.176)	0.149 (0.105)	0.209 (0.186)
	Week 12 (LOCF)	0.185 (0.276)	0.053 (0.067)	0.128 (0.106)	0.049 (0.049)
	% Change	29.9 (15.7, 44.2)	−62.3 (−70.5, −54.1) ***	−0.8 (−18.3, 16.6)	−65.9 (−79.0, −52.8) ***
VLDL-C	*n*	141	428	33	58
(mmol/L)	Baseline	1.312 (0.400)	1.312 (0.377)	1.327 (0.355)	1.397 (0.394)
	Week 12 (LOCF)	1.260 (0.447)	0.918 (0.306)	1.316 (0.380)	0.968 (0.348)
	% Change	−0.8 (−4.4, 2.9)	−27.4 (−29.5, −25.3) ***	0.5 (−6.4, 7.4)	−29.2 (−34.4, −24.0) ***
Large LDL-C	*n*	141	428	33	58
(mmol/L)	Baseline	0.487 (0.157)	0.494 (0.157)	0.503 (0.117)	0.485 (0.146)
	Week 12 (LOCF)	0.491 (0.160)	0.701 (0.184)	0.534 (0.164)	0.704 (0.177)
	% Change	3.3 (−2.4, 9.1)	50.7 (47.4, 54.0) ***	8.9 (−3.3, 21.2)	53.8 (44.6, 63.1) ***
Medium LDL-C	*n*	141	428	33	58
(mmol/L)	Baseline	0.970 (0.285)	0.975 (0.281)	0.994 (0.251)	0.957 (0.285)
	Week 12 (LOCF)	0.954 (0.280)	1.128 (0.268)	1.018 (0.255)	1.136 (0.322)
	% Change	−0.2 (−5.1, 4.7)	23.2 (20.4, 26.0) ***	5.9 (−4.4, 16.3)	24.9 (17.1, 32.7) **
Small LDL-C	*n*	141	428	33	58
(mmol/L)	Baseline	0.641 (0.191)	0.644 (0.182)	0.643 (0.147)	0.644 (0.189)
	Week 12 (LOCF)	0.618 (0.177)	0.576 (0.170)	0.640 (0.154)	0.577 (0.203)
	% Change	−1.8 (−6.1, 2.5)	−6.0 (−8.5, −3.5)	1.5 (−6.7, 9.7)	−7.8 (−14.0, −1.7)
Very small LDL-C	*n*	141	428	33	58
(mmol/L)	Baseline	0.269 (0.082)	0.275 (0.081)	0.272 (0.083)	0.274 (0.081)
	Week 12 (LOCF)	0.258 (0.072)	0.231 (0.070)	0.272 (0.094)	0.229 (0.084)
	% Change	−2.2 (−5.9, 1.6)	−12.5 (−14.7, −10.4) ***	1.4 (−6.2, 8.9)	−14.6 (−20.3, −8.9) **
Very large HDL-C	*n*	141	428	33	58
(mmol/L)	Baseline	0.055 (0.017)	0.056 (0.018)	0.054 (0.017)	0.056 (0.016)
	Week 12 (LOCF)	0.053 (0.017)	0.053 (0.017)	0.057 (0.017)	0.053 (0.019)
	% Change	−1.8 (−4.8, 1.2)	−3.2 (−5.0, −1.5)	7.2 (1.0, 13.5)	−4.3 (−9.0, 0.4) **
Large HDL-C	*n*	141	428	33	58
(mmol/L)	Baseline	0.146 (0.076)	0.150 (0.078)	0.138 (0.066)	0.148 (0.074)
	Week 12 (LOCF)	0.145 (0.075)	0.135 (0.083)	0.152 (0.069)	0.132 (0.084)

	% Change	2.1 (−2.6, 6.8)	−9.7 (−12.4, −7.0) ***	12.1 (1.5, 22.7)	−10.3 (−18.3, −2.3) **
Medium HDL-C (mmol/L)	*n*	141	428	33	58
	Baseline	0.366 (0.109)	0.366 (0.104)	0.338 (0.077)	0.343 (0.092)
	Week 12 (LOCF)	0.366 (0.114)	0.435 (0.135)	0.349 (0.068)	0.429 (0.126)
	% Change	0.8 (−2.6, 4.2)	19.9 (17.9, 21.8) ***	4.6 (−3.3, 12.4)	26.8 (20.8, 32.7) ***
Small HDL-C (mmol/L)	*n*	141	428	33	58
	Baseline	0.380 (0.079)	0.376 (0.073)	0.363 (0.066)	0.365 (0.065)
	Week 12 (LOCF)	0.380 (0.078)	0.470 (0.083)	0.365 (0.061)	0.484 (0.080)
	% Change	1.3 (−1.5, 4.1)	26.7 (25.1, 28.3) ***	1.3 (−5.0, 7.5)	34.9 (30.1, 39.6) ***
Very small HDL-C (mmol/L)	*n*	141	428	33	58
	Baseline	0.171 (0.035)	0.174 (0.034)	0.170 (0.043)	0.170 (0.036)
	Week 12 (LOCF)	0.172 (0.036)	0.204 (0.038)	0.172 (0.042)	0.208 (0.037)
	% Change	1.1 (−1.9, 4.1)	19.4 (17.7, 21.1) ***	2.5 (−3.9, 8.9)	25.2 (20.4, 30.0) ***

Data are presented as mean (standard deviation) for baseline and week 12 (LOCF), and least square means (95% confidence interval) for % change or change. * $p < 0.05$, ** $p < 0.01$, *** $p < 0.001$ vs. placebo by ANCOVA with baseline as covariant. [1] $eGFR_{male} = 194 \times sCr^{-1.094} \times age^{-0.287}$, $eGFR_{female} = 194 \times sCr^{-1.094} \times age^{-0.287} \times 0.739$. HPLC, high-performance liquid chromatography; FAS, full analysis set; eGFR, estimated glomerular filtration rate; CM-C, chylomicron-cholesterol; VLDL-C, very-low-density lipoprotein-cholesterol; LDL-C, low-density lipoprotein-cholesterol; HDL-C, high-density lipoprotein-cholesterol; LOCF, last observation carried forward; sCr, serum creatinine.

Table S4. Changes in safety parameters with concomitant statin treatment from baseline to week 12 (SAS).

**Parameter**		Baseline eGFR [1] ≥ 60 mL/min/1.73 m^2		Baseline eGFR [1] < 60 mL/min/1.73 m^2	
		Placebo	Pemafibrate 0.1–0.4 mg/day	Placebo	Pemafibrate 0.1–0.4 mg/day
sCr (mg/dL)	*n*	141	425	33	58
	Baseline	0.75 (0.14)	0.76 (0.13)	1.03 (0.18)	1.05 (0.16)
	Week 12	0.76 (0.14)	0.79 (0.15)	1.01 (0.19)	1.06 (0.19)
	Change	0.00 (−0.01, 0.01)	0.03 (0.02, 0.03) ***	−0.02 (−0.05, 0.01)	0.01 (−0.02, 0.03)
eGFR [1] (mL/min/1.73 m^2)	*n*	141	425	33	58
	Baseline	82.4 (15.6)	81.4 (14.6)	52.7 (7.1)	52.8 (5.5)
	Week 12	82.1 (15.5)	78.6 (14.7)	54.3 (9.1)	52.8 (7.0)
	Change	−0.2 (−1.3, 1.0)	−2.8 (−3.5, −2.2) ***	1.6 (−0.3, 3.5)	0.0 (−1.4, 1.4)
CK (U/L)	*n*	141	425	33	58
	Baseline	136.7 (73.9)	135.6 (103.8)	181.8 (253.4)	133.0 (81.0)
	Week 12	129.3 (62.3)	143.9 (128.1)	127.3 (46.3)	141.5 (77.0)
	Change	−6.8 (−24.8, 11.1)	8.1 (−2.2, 18.5)	−26.9 (−49.6, −4.1)	−7.2 (−24.3, 9.9)
AST (U/L)	*n*	136	418	33	58
	Baseline	31.5 (10.3)	31.9 (14.1)	30.0 (8.5)	28.5 (12.2)
	Week 12	32.6 (12.1)	30.7 (11.8)	27.6 (7.4)	30.6 (17.5)

	Change	0.9 (−0.7, 2.5)	−1.2 (−2.1, −0.2) *	−2.5 (−5.5, 0.4)	2.2 (−0.1, 4.4) *
ALT (U/L)	*n*	141	425	33	58
	Baseline	39.7 (18.6)	39.3 (21.1)	32.5 (13.9)	31.0 (15.1)
	Week 12	41.3 (21.8)	30.3 (19.1)	29.0 (11.5)	26.2 (15.3)
	Change	1.7 (−0.8, 4.1)	−9.0 (−10.5, −7.6) ***	−3.2 (−6.4, 0.1)	−5.0 (−7.5, −2.5)
γ-GT (U/L)	*n*	141	425	33	58
	Baseline	92.3 (97.8)	84.2 (81.7)	52.4 (36.2)	58.3 (38.2)
	Week 12	97.8 (114.1)	45.3 (44.6)	51.4 (38.1)	36.3 (37.3)
	Change	7.8 (0.8, 14.7)	−39.6 (−43.6, −35.6) ***	−2.2 (−11.6, 7.3)	−21.3 (−28.4, −14.3) **
ALP (U/L)	*n*	141	425	33	58
	Baseline	230.3 (60.3)	237.1 (67.7)	238.4 (66.6)	246.5 (79.8)
	Week 12	229.4 (62.6)	160.8 (52.7)	231.8 (62.5)	165.1 (53.8)
	Change	−2.5 (−7.7, 2.7)	−75.8 (−78.8, −72.7) ***	−8.5 (−18.9, 2.0)	−80.3 (−88.2, −72.5) ***
Total bilirubin (mg/dL)	*n*	141	425	33	58
	Baseline	0.78 (0.37)	0.77 (0.33)	0.74 (0.34)	0.70 (0.22)
	Week 12	0.80 (0.32)	0.64 (0.22)	0.74 (0.30)	0.61 (0.16)
	Change	0.02 (−0.01, 0.05)	−0.13 (−0.15, −0.11) ***	0.01 (−0.04, 0.07)	−0.10 (−0.14, −0.06) **

Data are presented as mean (standard deviation) for baseline and week 12, and least square means (95% confidence interval) for % change or change. * $p < 0.05$, ** $p < 0.01$, *** $p < 0.001$vs. placebo by ANCOVA with baseline as covariant. [1] $eGFR_{male} = 194 \times sCr^{-1.094} \times age^{-0.287}$, $eGFR_{female} = 194 \times sCr^{-1.094} \times age^{-0.287} \times 0.739$. SAS, safety analysis set; eGFR, estimated glomerular filtration rate; sCr, serum creatinine; CK, creatine kinase; AST, aspartate aminotransferase; ALT, alanine aminotransferase; γ-GT, gamma-glutamyltransferase; ALP, alkaline phosphatase.

Table S5. Summary of six placebo-controlled, double-blind, randomized trials of pemafibrate.

Study No.	Dose	Inclusion criteria regarding lipid parameters	Primary outcomes	*n* [1]	Duration	Reference
K-877-04<br>Dose finding	Placebo<br>Pemafibrate 0.05, 0.1, 0.2, or 0.4 mg/day<br>Fenofibrate 100 mg/day	TG: 2.26-5.65 mmol/L (200–500 mg/dL)<br>HDL-C:<br>< 1.29 mmol/L (50 mg/dL) (Male),<br>< 1.42 mmol/L (55 mg/dL) (Female)	Efficacy: Percent change in fasting TG<br>Safety: Incidence of AEs and ADRs	224	12 weeks	Ishibashi S, et al. Atherosclerosis. 2016; 249:36–43.
K-877-09<br>Compared to fenofibrate	Placebo<br>Pemafibrate 0.1, 0.2, or 0.4 mg/day<br>Fenofibrate 100 or 200 mg/day	TG: 2.26-11.29 mmol/L (200–1000 mg/dL)<br>HDL-C:<br>< 1.29 mmol/L (50 mg/dL) (Male),<br>< 1.42 mmol/L (55 mg/dL) (Female)	Efficacy: Percent change in fasting TG<br>Safety: Incidence of AEs and ADRs	526	12 weeks	Arai H, et al. J Atheroscler Thromb. 2018; 25: 521-538.
K-877-13<br>Add-on to pitavastatin	Placebo<br>Pemafibrate 0.1, 0.2, or 0.4 mg/day	TG: 2.26-11.29 mmol/L (200-1000 mg/dL)<br>Non-HDL-C: ≥ 3.88 mmol/L (150 mg/dL)	Efficacy: Percent change in fasting TG<br>Safety: Incidence of AEs and ADRs	188	12 weeks	Arai H, et al. Atherosclerosis. 2017; 261:144–152.
K-877-15<br>Add-on to any statin	Placebo<br>Pemafibrate 0.2 or 0.2 (0.4) [2] mg/day	TG: 2.26-11.29 mmol/L (200–1000 mg/dL)	Efficacy: Percent change in fasting TG<br>Safety: Incidence of AEs and ADRs	423	24 weeks	Arai H, et al. Atherosclerosis. 2017; 261:144–152.
K-877-16<br>Type 2 diabetes	Placebo [3]<br>Pemafibrate 0.2 or 0.4 mg/day	TG: 1.69-11.29 mmol/L (150–1000 mg/dL)	Efficacy: Percent change in fasting TG<br>Safety: Incidence of AEs and ADRs	166	24-52 weeks	Araki E, et al. Diabetes Care. 2018;41:538-546.
K-877-19<br>Glucose clamp	Placebo<br>Pemafibrate 0.4 mg/day	TG: 2.26-5.65 mmol/L (200–500 mg/dL)	Efficacy: Change in splanchnic glucose uptake<br>Safety: Incidence of AEs and ADRs	27	12 weeks	Matsuba I, et al. J Diabetes Investig. 2018; 9: 1323–1332.

[1] the number of randomized patients, [2] up-titrating from pemafibrate 0.2 mg/day to 0.4 mg/day after week 12 if TG levels ≥ 1.69 mmol/L (150 mg/dL) at week 8, [3] switching from placebo to pemafibrate 0.2 mg/day after week 24. TG, triglyceride; HDL-C, high-density lipoprotein-cholesterol; AEs, adverse events; ADRs, adverse drug reactions.

International Journal of
Molecular Sciences
